# A New RF Energy Harvesting System Based on Two Architectures to Enhance the DC Output Voltage for WSN Feeding

**DOI:** 10.3390/s22093576

**Published:** 2022-05-07

**Authors:** Chemseddine Benkalfate, Achour Ouslimani, Abed-Elhak Kasbari, Mohammed Feham

**Affiliations:** 1 Quartz Laboratory, Department of Electrical and Electronic Engineering, Ecole Nationale Supérieure de l’Electronique et de ses Applications, 95014 Cergy, France; achour.ouslimani@ensea.fr (A.O.); abed-elhak.kasbari@ensea.fr (A.-E.K.); 2 STIC Laboratory, Department of Telecommunications, Faculty of Technology, University Abou Bekr Belkaid, Tlemcen BP 230-13000, Algeria; mohammed.feham@univ-tlemcen.dz

**Keywords:** RF energy harvesting system, multi-bands antenna, patch antenna, rectifier, impedance matching circuit, rectenna, switch

## Abstract

In this paper, a new RF Energy Harvesting (RF-EH) system for Wireless Sensor Network (WSN) feeding is proposed. It is based on two different monitored architectures using switch circuits controlled by the input powers. One architecture is more adapted to high input powers and the other to low input powers. The two different architectures and the system are designed and realized on Teflon glass substrate with a relative permittivity of 2.1 and thickness of 0.67 mm. They are tested separately as a function of the distance from the relay antenna. A new multiband antenna with a size of 40 × 30 mm^2^ is used for both architectures and the system. The measured antenna gains are 2.7 dB, 2.9 dB, and 2.55 dB for the frequencies of 1.8 GHz, 2.1 GHz, and 2.66 GHz corresponding to the mobile communication networks, respectively. The rectifier consists of two Schottky diodes forming a full-wave rectifier and voltage doubler. The maximum measured RF-to-DC conversion efficiency is 71.5%. The proposed RF-EH system provides a maximum DC output voltage of 5.6 V and 3.15 V for an open and 2 kΩ resistance load, respectively.

## 1. Introduction

RF Energy Harvesting (RF-EH) applications are the focus of several researchers around the world. Several techniques have been proposed to ultimately solve the problem of battery life and ensure the energy autonomy of electronic equipment.

**Figure 1:**
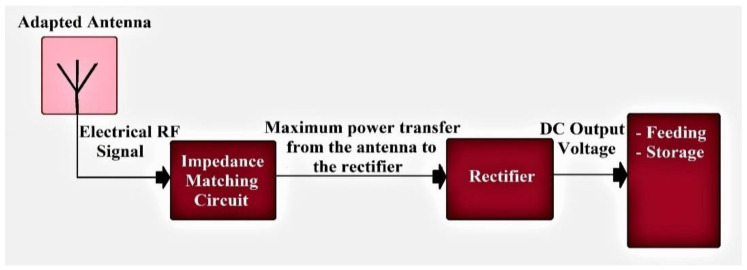
RF Energy Harvesting system blocks.

RF-EH systems consist of an antenna, impedance matching circuit, and a rectifier as presented in Figure 1. 

Several researchers propose many architectures to enhance the output voltages. Multi-stages Dickson voltage multiplier circuits based on Schottky diodes [1,2,3] or CMOS transistors [4,5] are proposed. The use of many active components decreases the efficiency of RF-EH systems due to the energy consumption and leakages. In [6,7,8,9], the authors propose a system in which the antenna is followed by a power divider circuit. Each output port of the divider is matched to a rectifier for a given frequency and all the DC output voltages of the rectifiers are then combined. This architecture, which divides the RF power collected by the antenna to several rectifiers, reduces the efficiency of the energy harvesting system. In addition, to maintain sufficient efficiency, this architecture requires a large antenna, which is contrary to the miniaturization.

**Figure 2:**
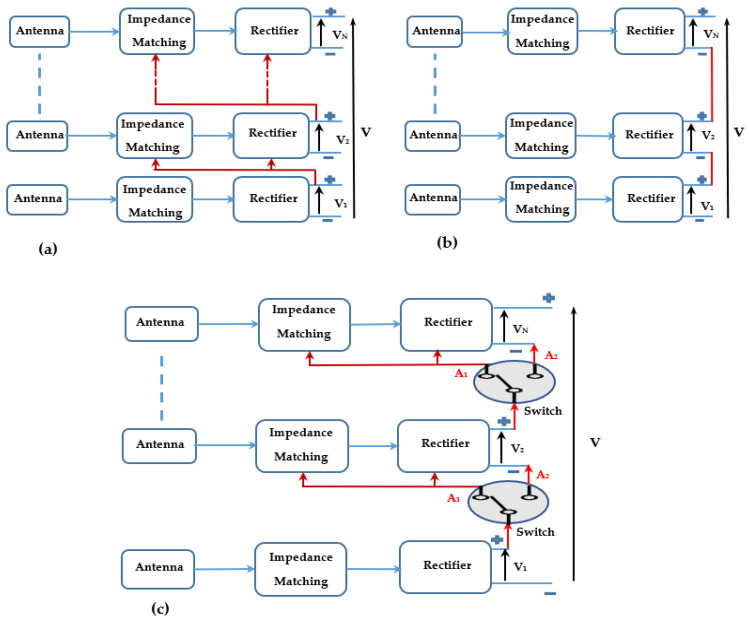
Block diagram of (**a**) first architecture (RF-EH1), (**b**) second architecture (RF-EH2), and (**c**) the proposed system RF-EH. A_1_ and A_2_ are the two positions corresponding to first and second architectures.

In this paper, we propose a new RF-EH system based on two different architectures. One architecture (Figure 2a) is more adapted to low input powers and the other to high input powers (Figure 2b). The proposed system uses switches to select the more suitable architecture (Figure 2c) according to the input power. Both architectures and the system have been designed and realized on Teflon glass substrate hybrid technology and tested as a function of the distance from the mobile communication base station.

Generally speaking, the architectures consist in exploiting N rectennas (antenna + rectifier) that are matched to the N frequency bands that exist in the environment. 

**Figure 3:**
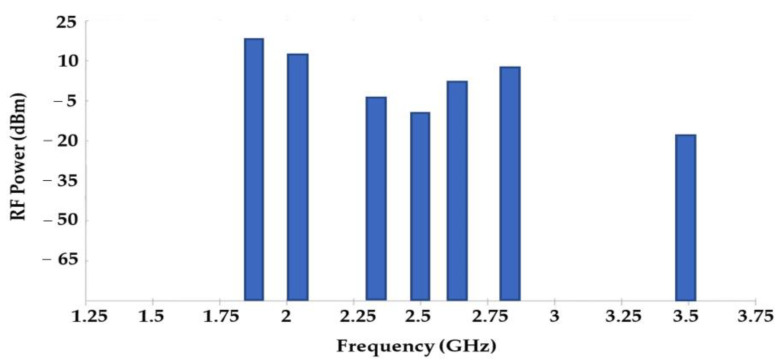
Measured ambient RF power at 22 m from mobile communication base station.

In this work, three rectennas are used to demonstrate the feasibility of the system. These rectennas are matched to the most powerful mobile communication network bands (GSM, UMTS, LTE-2.6) as presented in Figure 3. 

The first architecture, presented in Figure 2a, uses the DC output voltage V_1_, provided by the rectenna-1 matched to the GSM network, to shift up the average value of the RF signal received by the rectenna-2, which is adapted to the UMTS network. The DC output voltage V_2_, supplied by the rectenna-2, is used to increase the average value of the RF signal received by the rectenna-3 that is matched to the LTE-2.6 network. 

The second architecture realizes a summation of the three DC output voltages provided by the three rectennas as depicted in Figure 2b. 

The proposed system (Figure 2c) uses two switches to move from one architecture to another depending on the input power levels. A switching zone is observed in the input power interval of [−6–−5.5] dBm. For low input powers, the system switches to the first architecture and switches to the second one for high input powers. 

The proposed system provides a maximum measured DC output voltage of 3.15 V at 22 m from the base station and a maximum efficiency of 71.5% at 75 m for a 2 kΩ resistance load. This system combines the advantages of both architectures. 

For each rectenna, the proposed antenna is a new multi-band one covering all the mobile communications and wireless network bands (GSM-1800, UMTS, LTE-2.3, Wi-Fi, and LTE-2.6). The choice of this multi-band antenna results in a compact size antenna, compared to a single mono-frequency antenna that only covers one of these bands. The dimension of the proposed antenna is 40 × 30 mm^2^. The rectifier is realized with two Schottky diodes (SMS7630-079LF) that form a full-wave rectifier and voltage doubler. This rectifier is matched to the antenna by using two LC resonators. Simulations were carried out using CST and ADS softwares.

**Figure 4:**
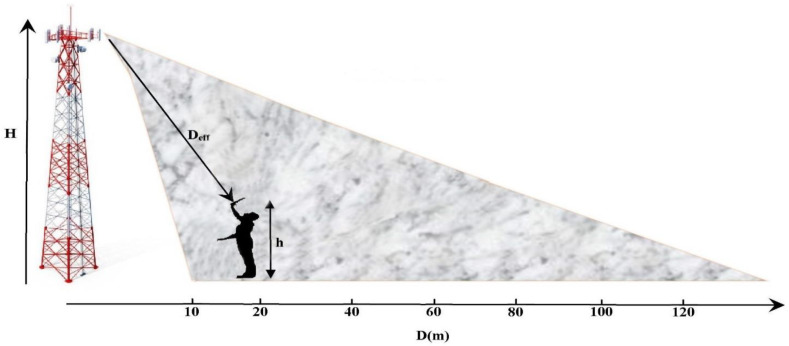
Geometric parameters of measurement field. D_eff_: Effective distance.

Figure 4 shows that the effective distance between the relay antenna and the system under test is given as a function of the height of the mobile communication relay antenna and other parameters. The effective height can be defined as the difference between the height of the relay antenna and that of the device under test.

The expression of the effective distance can be extracted by using Pythagorean law on the right triangle formed by the effective height and the horizontal distance between the relay antenna and the measuring point, as given by the Equation (2).

(1) $$H_{eff}=H-h=21.7-2=19.7\;m$$

(2) $$D_{eff}=\sqrt{D^{2}+H_{eff}{}^{2}}=\sqrt{D^{2}+388}$$

The coverage of this relay antenna starts from D = 10 m. Applying Equation (2), the effective distance is 22 m at this position. The measured received RF powers by the three rectennas (GSM-1800, UMTS, and LTE-2.6) are depicted in Table 1.

In the next paragraphs, we emphasize the antenna part, rectennas, both proposed architectures, and the proposed RF-EH system, respectively.

## 2. Stat of the Art on RF Energy Harvesting Systems

Many works on RF energy harvesting system architectures have been reported in the literature [10,11,12,13,14,15,16,17,18,19,20,21,22,23,24]. In these systems, different antenna and rectifier structures have been proposed to obtain sufficient RF output power with improved gain and efficiency. The rectifiers are connected to the antennas via impedance matching circuits. From these reported works, we can classify the RF-EH systems into five categories: the single-band, the broadband, the multi-band, the multi-port, and the reconfigurable RF-EH systems, which are highlighted in this section.

### 2.1. Single-Band RF-EH Systems

These systems are designed with a single band antenna with a full or half rectifier based on Schottky diodes [10,11,12]. In [10], the proposed system uses a single-band wire printed antenna realized by tow symmetric patches adapted to the 2.45 GHz frequency. The rectifier consists of two Schottky diodes (HSMS-2852) connected to the antenna by a coplanar strip-line. The rectifier is matched to the antenna using an impedance matching circuit designed by microstrip lines and open stubs. This system provides a maximum RF-to-DC conversion efficiency of 50% for a −17.2 dBm input power and 1.4 kΩ load resistance. In [11], the authors present a system with a flexible and multilayered (3 layers) antenna matched to the 2.45 GHz frequency. It demonstrates a maximum radiation efficiency of 62%. The flexibility of this antenna allows it to reduce the size of the RF-EH system. The system uses a full wave rectifier designed by Schottky diodes (SMS7630) and an impedance matching circuit based on microstrip lines with open stubs. It provides a maximum RF-to-DC conversion efficiency of 65% for a 0 dBm input power. In [12], the RF-EH system is proposed to exploit the provided RF power from the GSM-900 network. The used antenna covers a frequency bandwidth of (823.7–1336.9) MHz. It demonstrates a radiation efficiency of 80.8% and maximum gains of 2.96 dB and 3.62 for frequencies of 900 MHz and 1227 MHz, respectively. The RF-EH system provides a maximum RF-to-DC conversion efficiency of 61.7% for a −5 dBm input power. The matching of the antenna to the rectifier is ensured using an LC resonator as an impedance matching network.

### 2.2. Broad-Band RF-EH Systems

These systems use a broadband antenna followed by a matched rectifier(s). The matched rectifier circuit can be either a single broadband one [13,14] or many narrowband rectifiers connected to the outputs of RF power divider [15]. In [13], a wide-band circular patch antenna is reported. It covers a frequency bandwidth of [0.8–3.6] GHz. To exploit the full frequency band available at the output of the antenna, the authors propose a broadband matched rectifier circuit ranging from 1 GHz to 3.5 GHz. This RF-EH system provides a maximum RF-to-DC conversion efficiency of 73.4% for a 3 dBm input power. In [14], the authors adopt the same technique, but for lower frequencies ranging from 0.45 GHz to 1 GHz. This system demonstrates a maximum RF-to-DC conversion efficiency of 77% for a −1 dBm input power. In [15], the authors propose an RF-EH system in which an antenna covering a band of [0.9–2.6] GHz is connected to the RF power divider with two outputs. Each output of the divider is then connected to a matched rectifier circuit to realize either a multi-output RF energy harvesting system or to sum the output voltages for improved system performances. The two rectifiers are matched to 900 MHz and 2.45 GHz. The overall DC voltage obtained by this system at 400 m from a base station (relay antenna) is 243 mV for a 4.7 kΩ load resistance.

### 2.3. Multi-Band RF-EH Systems

In these systems, the antenna is a multi-band one followed either by a multi-band rectifier or several single-band rectifiers that are matched to the antenna for different frequencies [16,17,18,19]. In [16], the authors present a 3D RF energy harvesting system with a multi-band antenna covering the frequencies 1.8 GHz, 2.1 GHz, 2.45 GHz, and 2.6 GHz. It demonstrates a maximum gain of 10 dBi at 2.45 GHz. This antenna is connected to four rectifiers using a four-output RF power divider. The rectifiers are designed by two SMS7630 Schottky diodes. The maximum measured RF-to-DC conversion efficiency for each rectifier are 27%, 26%, 25.5%, and 27.5% for the four frequencies of 1.8 GHz, 2.1 GHz, 2.45 GHz, and 2.6 GHz, respectively, and a −6 dBm input power. A maximum voltage of 484 mV is provided by the rectifier matched to the 2.6 GHz frequency for a 3.3 kΩ load resistance. In [17], the proposed RF energy harvesting system uses a multi-band antenna with 12 output ports that are matched to bands of [1.7–1.8] GHz and [2.1–2.7] GHz. Each output port is connected to a full-wave rectifier based on SMS7630 Schottky diodes and matched to the antenna by open stubs. This system demonstrates a maximum RF-to-DC conversion efficiency of 67% for a −3 dBm input power. The output voltages of each rectifier are summed to improve the system performance. This provides maximum DC output powers of 65 µW and 20 µW outside and inside the building (Outdoor/Indoor) for ambient power densities of −13 dBm/cm^2^ and −40 dBm/cm^2^, respectively. In [18], the authors propose a dual-band system in which the antenna is a dual band one covering the UHF [855–935] MHz and ISM 2.45 GHz bands. The maximum radiation efficiencies and gains of this antenna are 88% and 92% and 2 dBi and 3.8 dBi for the UHF and ISM bands, respectively. The antenna is connected to two rectifiers using a two output RF power divider. One rectifier is matched to the UHF band while the other is matched to the ISM band. The maximum voltage delivered by this system is 0.7 V for an input power of −12 dBm and a 10 kΩ load resistance. The maximum RF-to-DC conversion efficiency is 65% for an input power of −5 dBm. in [19], the authors propose a dual band system. It uses a dual band antenna based on many resonators that are connected together using diodes to route the currents provided by each resonator to a single point and ensure a combination of the RF signals. The two bands covered by this antenna are ISM 2.45 GHz and ISM 5.8 GHz. The antenna is connected to a dual band matched rectifier covering the same bands as the antenna. The maximum RF-to-DC conversion efficiency provided by this system is 70% for a 10 dBm input power. This efficiency is obtained using the rectifier designed by the HSMS2860 Schottky diode.

### 2.4. Multi-Port RF-EH Systems

These systems use RF power combiners with two or more inputs [20,21,22]. The used multi-port antenna could be either a single band, multi-band, or broadband one. Each antenna port is then connected to the combiner and the output of the combiner is connected to the rectifier. In [20], the authors propose a complex structure (3D structure) for an RF-EH system that consists of a multi-band/multi-port antenna (8 ports) where each pair of ports is then connected to a 2-way to 1-way combiner (two inputs, one output). The output of each combiner is then connected to a rectifier based on HSMS-2850 Schottky diodes. This RF-EH system covers the GSM-900, GSM-1800, 3G (2.1 GHz), Wi-Fi (2.45 GHz), and LTE (2.6 GHz) bands. For a −20 dBm input power and 2.1 kΩ load resistance, the authors register maximum RF-to-DC conversion efficiencies of 66.52%, 51.5%, 56.5%, 31.9%, and 30% for the 3G (2.12 GHz), GSM 900, GSM 1800, Wi-Fi, and LTE bands, respectively. For a lower input power (−35 dBm), the efficiencies drop to 45%, 41.2%, 38.1%, and 34% for the summed outputs of GSM 900 + GSM 1800, GSM 900 + GSM 1800 + 3G, GSM 900 + GSM 1800 + 3G + Wi-Fi and GSM 900 + GSM 1800, + 3G, + Wi-Fi, and + LTE, respectively. The overall DC voltage collected is 0.75 V for an input power of −27 dBm and a 2.1 kΩ load resistance. In [21], the proposed RF-EH system consists of a single band multi-pixel antenna with 4 output ports. Each output of this antenna is connected to a Schottky diode HSMS-2850 full wave rectifier adapted for the 1.8 GHz frequency, which is the center frequency of the band covered by the antenna. The impedance matching circuit is realized using microstrip lines and shorted stubs. For each port, the RF-to-DC conversion efficiency is 11.9%, 33.4%, and 51.3% for −30 dBm, −20 dBm, and −10 dBm input powers, respectively. Numerous studies have been made to select one combination that allows for the achievement of a maximum power of 11.2 μW. In [22], the authors propose a multi-port RF-EH system that uses a single-band/multi-layer Grid Array Antenna (GAA) with dual output ports matched to the 2.45 GHz frequency. It allows it to obtain a high sensitivity at low input powers and has a wide incidence angle range. Each output port is connected to a half-wave rectifier designed by an SMS-7630 Schottky diode. The RF-EH system consists of two balanced rectifiers whose output DC powers are efficiently combined. One rectifier is connected to a 3 kΩ load resistance and the other to a 6 kΩ load resistance. The output DC power is greater than 200 μW for an incidence angle range of [−30° to 30°]. The maximum obtained RF-to-DC conversion efficiency is 45% for an input power density of 1 μW/cm^2^.

### 2.5. Reconfigurable RF-EH Systems

These systems are manufactured (designed) with a single band, a multiband, or a broadband antenna. The rectifier is matched to the antenna by using an impedance matching structure based on active components (Diodes or Transistors) that form a switch [23,24]. The system proposed in [23] is a reconfigurable adaptive system consisting of a single-band microstrip patch antenna and a reconfigurable rectifier circuit. To have better adaptation for variable input power, a reconfigurable adapter is deployed between the antenna and the rectifier that is flexibly adjusted. To miniaturize the circuit size, the matching section takes the shape of an L, where a PIN diode switch (SMP1345-SC-79) is in series to implement the reconfigurable matching section. Depending on the input power levels, the PIN switch states can be automatically monitored to disconnect/connect the matching stub from/to the rectifier circuit to achieve better matching performance at different power levels. The proposed system operates at 5.8 GHz for a wide input power range. In this design, a 150-pF pre-capacitor is used to protect the antenna from a reverse current. An HSMS 2850 Schottky diode with a low junction capacitance (C_j0_ = 0.18 pF) is connected in parallel to the rectifier circuit. Two radial microstrip stubs are used as a DC pass filter and serve to suppress high-order harmonics and smooth the output DC voltage waveform—in cooperation with the pre-capacitor. The measured results for a 220 Ω load resistance show that this RF-EH system achieves an RF-to-DC conversion efficiency of more than 50% for a wide input power range of 0 to 20 dBm, with a maximum efficiency of 68% at 9 dBm and a maximum DC output voltage of 3.6 V at a 20 dBm input power. 

The authors in [24] present a frequency reconfigurable RF-EH system. It is designed by a broadband patch antenna and a half-wave rectifier based on an HSMS-2850 Schottky diode. The frequency bandwidth covered by the antenna is [3.5–6.5] GHz. For the frequencies of 5.2 GHz and 5.8 GHz, the measured gains are 4.45 dBi and 4.49 dBi, respectively. The measured radiation efficiencies are 94% and 90.1%, respectively, for both frequencies. The rectifier is matched to the antenna for both frequencies of 5.2 GHz and 5.8 GHz by a reconfigurable shorted stub. Depending on the DC output voltage for both frequencies, an automatic ON-OFF of an adaptive FET switching is performed, and the stub is connected/disconnected to/from the rectifier circuit for a good matching between the antenna and the rectifier. For a 6.5 dBm input power and 200 Ω load resistance, the maximum obtained RF-to-DC conversion efficiencies are 54.9% and 52.2% for the 5.2 GHz and 5.8 GHz frequencies, respectively. 

Table 2 shows the substrates used to realize the RF-EH systems seen in this section.

## 3. Antenna Design and Measurements

**Figure 5:**
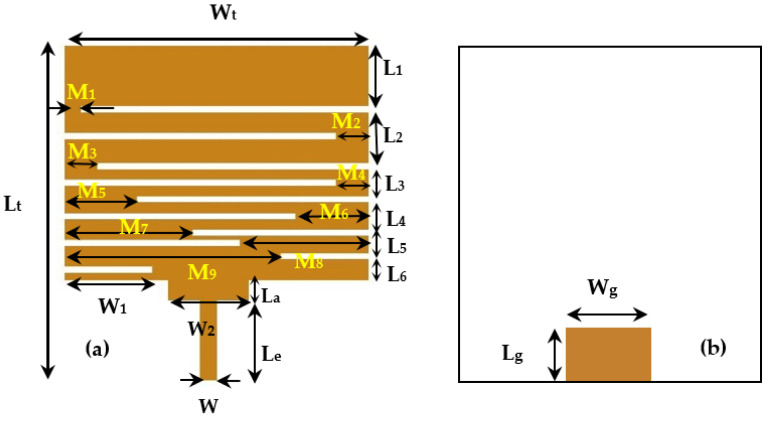
Proposed antenna structure, (**a**) Top side, (**b**) Bottom side.

**Figure 6:**
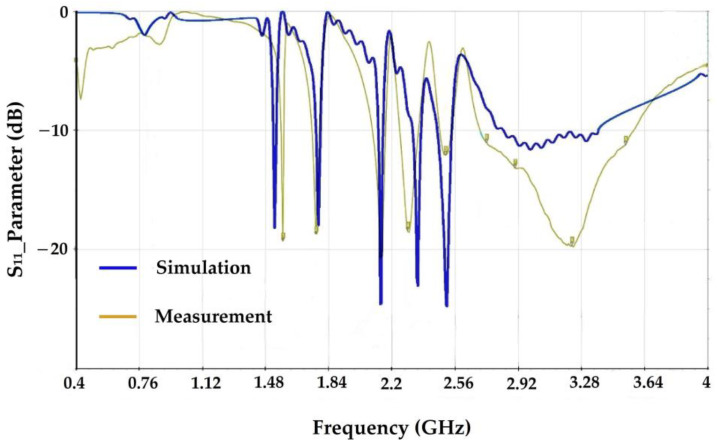
The simulated and measured S_11_ parameter of the proposed antenna.

The proposed antenna is a meander line one. Meander forms can be considered as deformed wires that create resonance effects. These resonators can either resonate at the same frequency, which allows them to enhance the antenna performance at this frequency, or resonate at different frequencies in order to obtain a multi-band antenna. Three-dimensional Electromagnetic simulations under CST software are carried out to optimize the antenna performances and dimensions. The proposed antenna consists of 8 meanders of different widths and lengths where each meander resonates at its own frequency, resulting in 8 frequencies covered by this antenna. The antenna is realized on Teflon glass substrate (ε_r_ = 2.1, thickness = 0.67 mm). The size of this antenna is 40 × 30 mm^2^. The input impedance is 50 Ω. Figure 5 shows the proposed antenna model. Table 3 gives all the antenna dimensions. Figure 6 depicts the simulated and measured S_11_ parameter of the antenna.

**Figure 7:**
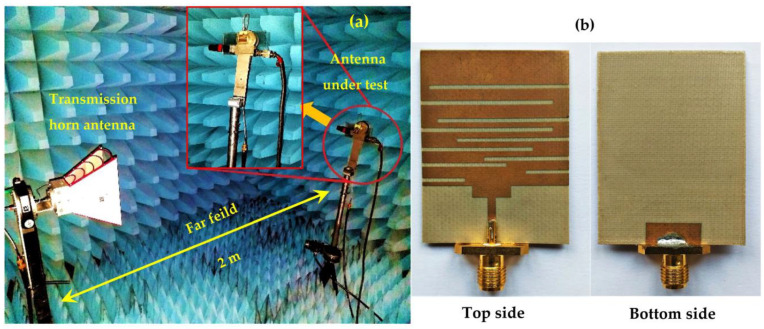
(**a**) Measurement prototype and (**b**) realized antenna.

The simulation and measurements are in good agreement. The measurements demonstrate that the antenna covers the frequencies of 1.8 GHz, 2.11 GHz, 2.3 GHz, 2.5 GHz, and the band from [2.6–3.6] GHz with S_11_ values of −18 dB, −21 dB, −18 dB, −13 dB, and −19 dB at the central frequency of the band, respectively. Figure 7 shows the realized antenna and measurement prototype. 

**Figure 8:**
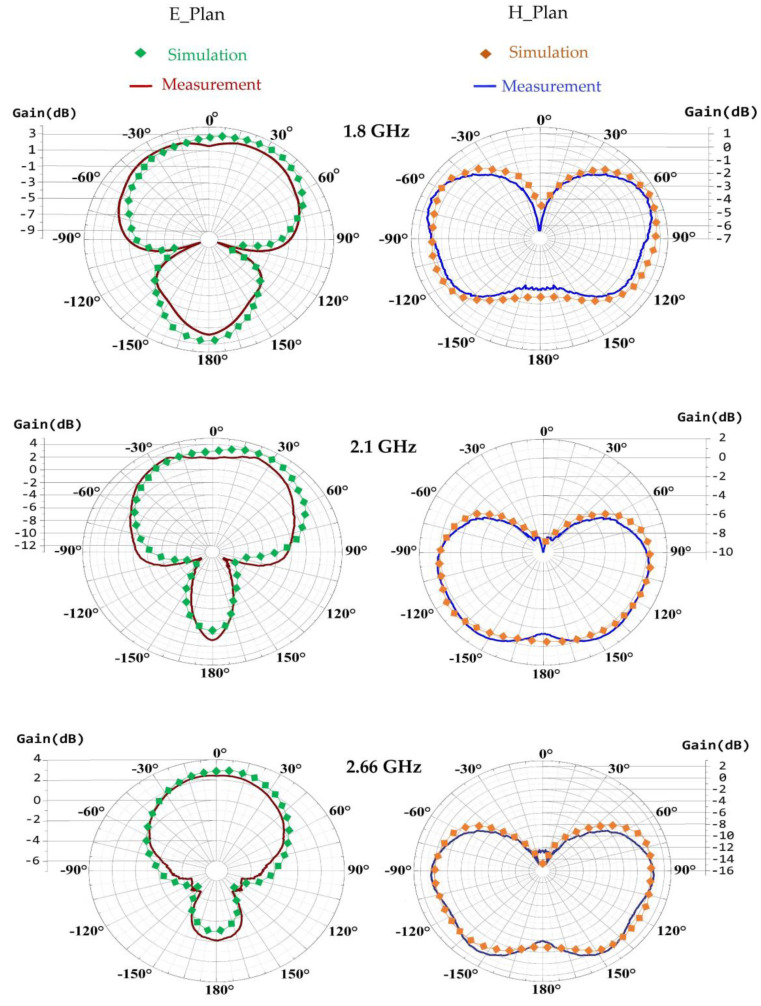
Simulated and measured radiation pattern for GSM, UMTS, and LTE bands, respectively.

Figure 8 depicts the simulated and measured radiation pattern versus gain in dB of the proposed antenna for the frequencies 1.8 GHz, 2.1 GHz, and 2.66 GHz, respectively.

It can be noted that the simulated and measured radiation pattern of this antenna are in good agreement and remain almost the same at the three frequencies of 1.8 GHz, 2.1 GHz, and 2.66 GHz with measured gain values of 2.7 dB, 2.9 dB, and 2.55 dB, respectively. The measured efficiencies are 70%, 65%, and 68.5% for 1.8 GHz, 2.1 GHz, and 2.66 GHz, respectively. All these results make this antenna suitable for RF-EH applications.

## 4. Rectenna Design and Measurements

**Figure 9:**
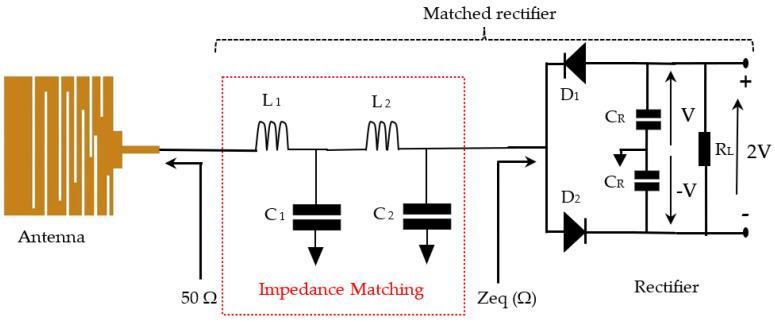
Rectenna circuit with ideal impedance matching, D_1_, D_2_: (Schottky diodes: SMS7630-079LF), C_R_ = 470 uF, R_L_ = 2 kΩ.

Figure 9 depicts the rectenna, which is the association of the antenna, rectifier, and impedance matching circuit in which inductors and capacitors are ideal. The rectifier is formed by two Schottky diodes and two capacitors forming a DC volage doubler. The impedance matching circuit is based on two L, C resonators (L_1_, C_1_ and L_2_, C_2_). Three rectennas are designed and realized for the GSM-1800, UMTS (3G), and LTE-2.6 bands.

For each band, the structure of the rectenna remains the same, only the values of the electrical elements (L_1_, C_1_ and L_2_, C_2_) of the impedance matching circuit change with the frequency. Table 4 gives the values of the electrical components of the impedance matching circuit for 1.8 GHz, 2.11 GHz, and 2.66 GHz.

**Figure 10:**
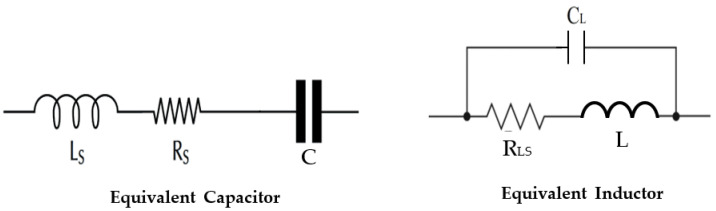
Equivalent circuit of capacitor and inductor. L_s_: Series inductance, R_s_: Electrodes and terminations resistance, C: Capacitance, L: Inductor, C_L_: Parasitic capacitor, R_LS_: Intern resistor.

In practice, the capacitors and inductors are a complex component combining resistive, inductive, and capacitive phenomena as shown in Figure 10. 

**Figure 11:**
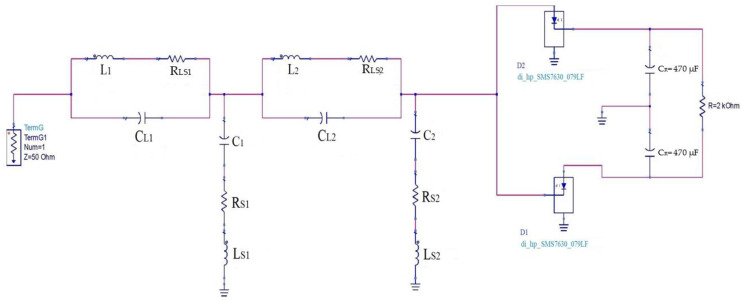
Practical rectenna circuit with all parasitic elements.

The quality factors Q_L_ and Q_C_ values of each component are given in their datasheet. The other element values could be derived using the resonance condition and quality factor expressions for both components (capacitor and inductor), as is given in Equations (3)–(5). Figure 11 depicts the practical rectenna circuit with all the parasitic elements. Table 5 gives all the parasitic element values.

(3) $$\omega_{0}{}^{2}=\frac{1}{\mathrm{LC}}$$

(4) $$Q_{L}=\frac{{L\omega }_{0}}{R_{\mathrm{LS}}}$$

(5) $$Q_{C}=\frac{1}{R_{S}{C\omega }_{0}}$$

**Figure 12:**
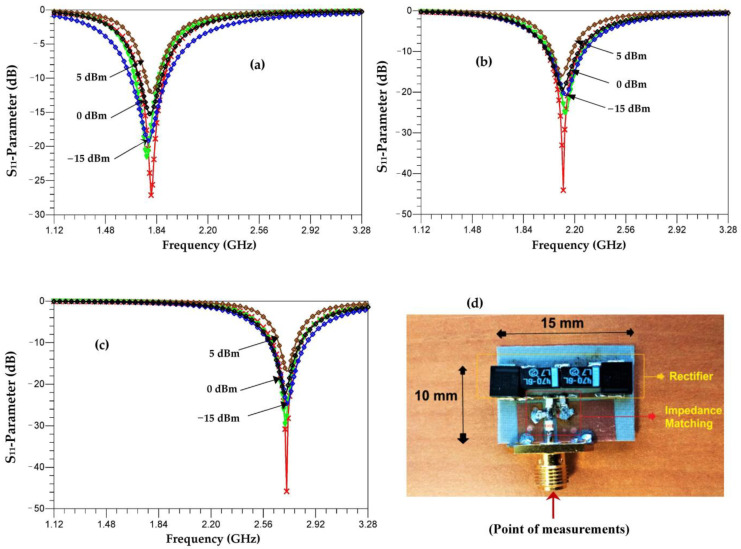
Simulated and measured S_11_ parameters of the three rectifiers for each frequency bands ((**a**) 1.8 GHz, 2.1 GHz (**b**) and 2.66 GHz (**c**)) and realized prototype (**d**). 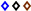 Measurement, 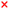 Simulation 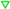 Co-simulation with parasitic elements.

Figure 12 depicts the simulated, co-simulated, and measured S_11_ parameters of the three matched rectifiers (rectifier + impedance matching) for the three bands of 1.8 GHz, 2.1 GHz, and 2.66 GHz. In the co-simulation part, we take into consideration the parasitic elements of the impedance matching circuit. The simulation and co-simulation are performed using ADS software for a −15 dBm input power. At −15 dBm, a good correlation between measurement and co-simulation is obtained. The matching level varies with the input power such that each time the power is increased, the S_11_ degrades with a small offset for the three resonance frequencies. In general, it can be observed that the measured S_11_ deeps of the prototype are at the desired frequency operation range for the GSM-1800, UMTS, and LTE bands, thereby allowing the rectenna to operate in all three bands for this order of input powers.

**Figure 13:**
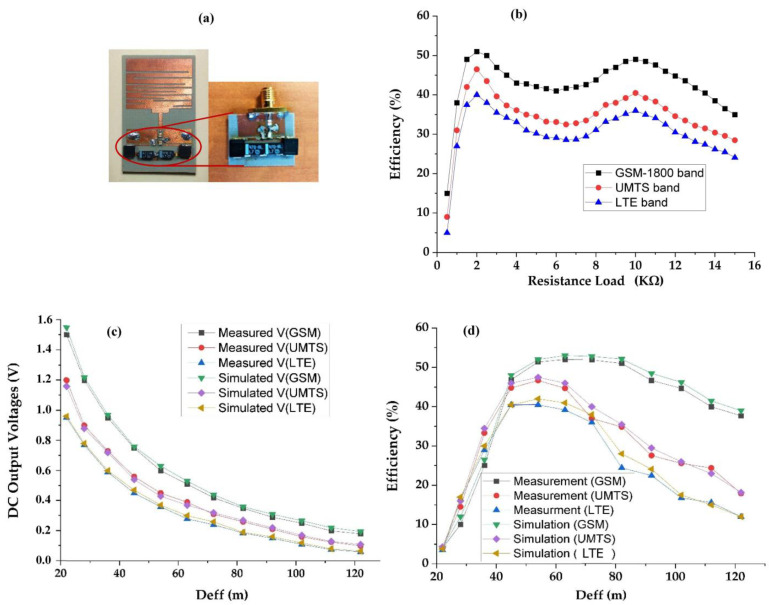
(**a**) Realized rectenna prototype, (**b**) Efficiency variation versus resistance load at 54 m, and (**c**,**d**) simulated and measured DC output voltages and RF-to-DC conversion efficiency, respectively, for GSM-1800, UMTS, and LTE-2.6 bands for 2 kΩ resistance load.

The realized rectenna prototype, which simulated and measured the DC output voltages and RF-to-DC conversion efficiencies for the three rectennas matched to the GSM-1800, UMTS, and LTE-2.6 bands, respectively, are depicted in Figure 13. The RF-to-DC conversion efficiency can be calculated by using Equation (6):

(6) $$\eta \left(\%\right)=\frac{P_{out}}{P_{int}}\cdot 100=\frac{V_{DC}^{2}}{R_{L}\cdot P_{int}}\cdot 100$$

where $P_{int}$, $P_{out}$, $R_{L}$,$V_{DC}$ are the input RF power, output DC power, resistance load, and the DC output voltage, respectively.

Figure 13b presents efficiency as a function of the load resistance. It can be seen that the maximum efficiency is obtained for a load resistance of 2 kΩ.

Figure 13 shows a good agreement between the simulations and measurements. At an effective distance of 60 m from the relay antenna, the rectenna matched to the GSM-1800 network has a maximum simulated and measured RF-to-DC efficiency of 53% and 52%, respectively. At this distance, the measured harvested DC power is 180 μW. At a distance of 54 m, the rectenna adapted to the UMTS network has a maximum simulated and measured RF-to-DC conversion efficiency of 47.5% and 46.7%, respectively. At this position, the measured harvested DC power is 101 μW. The rectenna that is adapted to the LTE-2.6 network has a simulated and measured RF-to-DC conversion efficiency of 42% and 40.5%, respectively, at 54 m from the relay antenna. At this distance, this rectenna provides a harvested DC power of 64.8 μW. 

At 122 m, the measured RF-to-DC conversion efficiencies are 37.7%, 17.9%, and 12%, and the measured DC output powers are 16.2 μW, 5 μW, and 0.18 μW for GSM-1800, UMTS, and LTE-2.6, respectively.

## 5. Proposed Architectures to Enhance the Performances of the RF Energy Harvesting Systems

In order to enhance the performance of the RF energy harvesting system in terms of DC output power as well as RF-to-DC efficiency even far from relay antenna (low input powers) two architectures have been designed, simulated, and tested.

The efficiency of the architectures are given by Equation (7):

(7) $$\eta \left(\%\right)=\frac{P_{out}}{P_{GSM}+P_{UMTS}+P_{LTE}}\cdot 100=\frac{V_{DC}^{2}}{R_{L}\cdot (P_{GSM}+P_{UMTS}+P_{LTE})}\cdot 100$$

where $P_{out}$, $R_{L}$,$V_{DC}$ are the input RF power, output DC power, resistance load, and the DC output voltage, respectively.

### 5.1. First Architecture (RF-EH_1_)

**Figure 14:**
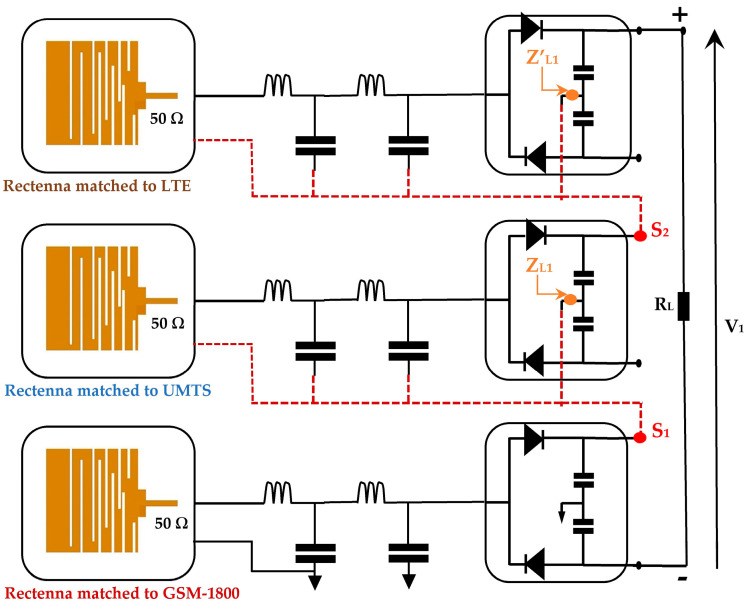
First architecture with three rectennas matched to GSM-1800, UMTS, and LTE-2.6. Z_L1_, Z’_L1_ are the impedances at the selected points.

This architecture consists of a superposition of the three rectennas studied previously. Due to the higher transmission power of the base station for the GSM-1800 network, the rectenna adapted to GSM-1800 must be placed in the first position to shift the RF signals of the following rectenna by a large DC component, which allows the diodes to operate in their stable state. In this case, the losses at the diodes become negligible for a certain input power range. Points S_1_ and S_2_ become the references for the second and third rectenna, respectively, as shown in Figure 14.

The impedances Z_L1_ and Z’_L1_ are characterized using ADS software (data items). The real parts are equal to 7.2 kΩ and 8.13 kΩ, respectively, with a negligeable imaginary one.

**Figure 15:**
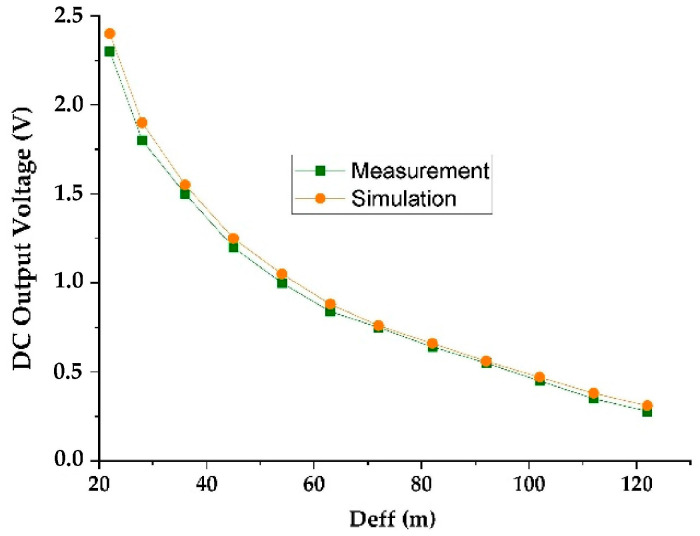
Simulated and measured DC output voltage for 2 kΩ resistance load.

**Figure 16:**
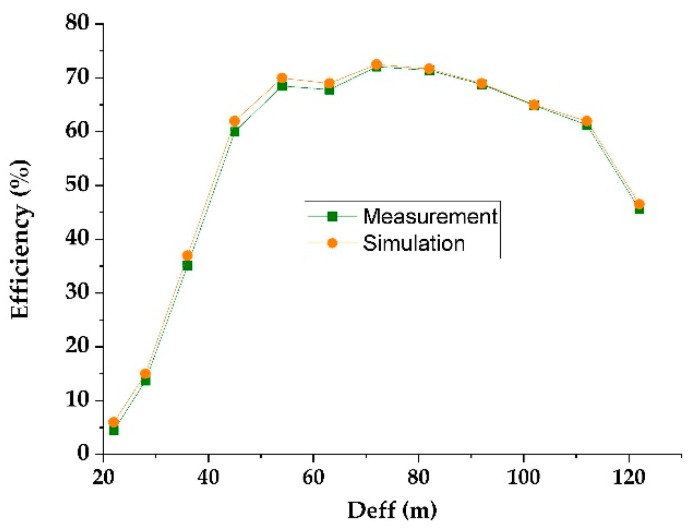
Simulated and measured RF-to-DC conversion efficiency for 2 kΩ resistance load.

Figure 15 shows the simulated and measured DC output voltages as a function of the effective distance between the RF-EH_1_ and the mobile communication relay antenna. Figure 16 presents the variation in the simulated and measured RF-to-DC conversion efficiency.

The maximum simulated and measured efficiencies of 71.5% and 71% are achieved at 72 m from the mobile communication relay antenna. These efficiencies stay above 50% until 122 m. The measured DC power supplied by this architecture is 288.8 μW at the effective distance of 72 m and 39.2 μW at 122 m. These results demonstrate that this architecture is suitable for low input powers (far distances from relay antenna) and limited for high input powers (near to relay antenna). 

**Figure 17:**
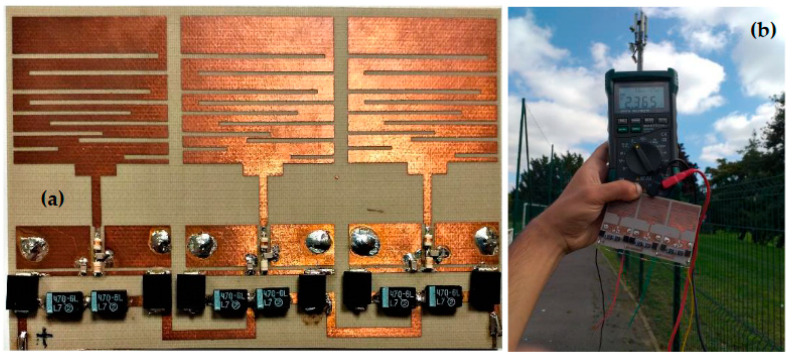
(**a**) Realized RF-EH1 based on the first architecture, (**b**) Measurement prototype at 22 m from relay antenna.

Figure 17 shows the realized RF-EH_1_ with a dimension of 64 × 55 mm^2^.

### 5.2. Second Architecture (RF-EH_2_)

**Figure 18:**
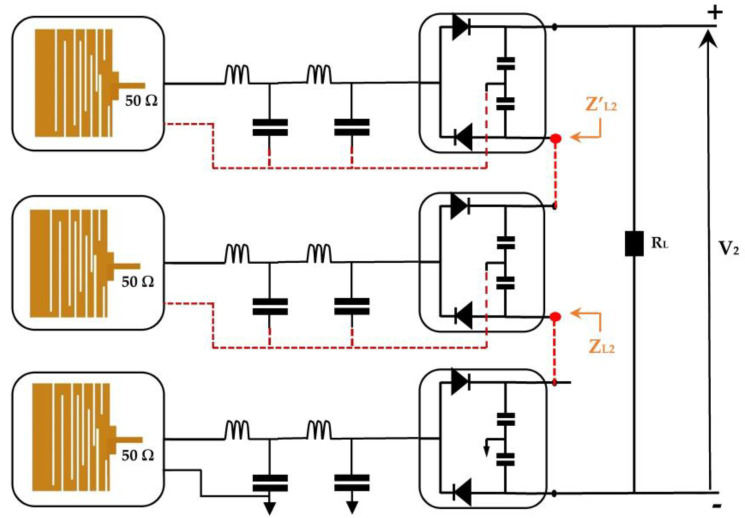
Second architecture with three rectennas matched to GSM-1800, UMTS, and LTE-2.6. Z_L2_, Z’_L2_ are the impedances at the selected points.

Figure 18 presents the second architecture. It consists of summing the DC output voltages delivered by each rectenna (antenna + rectifier) [14,15,16,25]. This architecture presents an ideal summation when the system is an open load. The presence of the load degrades it’s performance in terms of the DC output power. 

**Figure 19:**
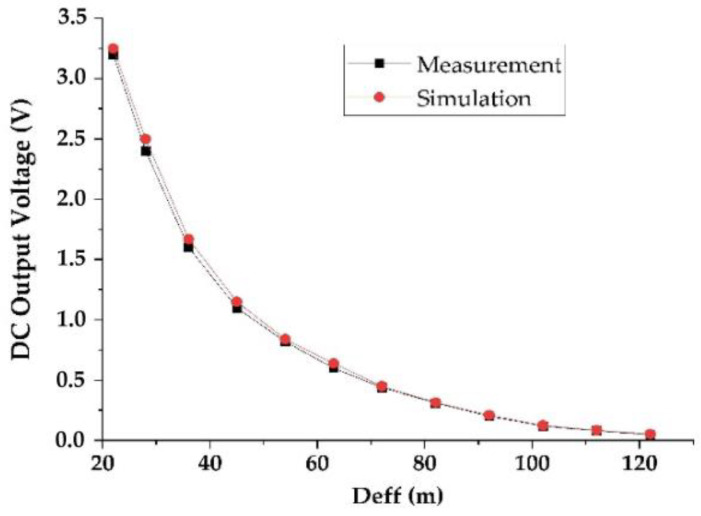
Measured and simulated DC output voltage as a function of the effective distance for 2 kΩ resistance load.

**Figure 20:**
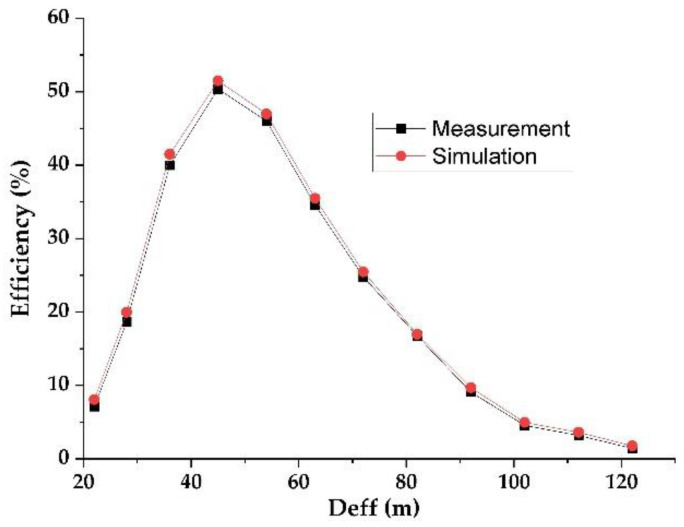
Measured and simulated efficiencies provided by the second architecture for 2 kΩ resistance load.

The impedances Z_L2_ = 0.1 + j0.0015 and Z’_L2_ = 0.095 − j0.0084 are characterized using ADS software (data items). Figure 19 and Figure 20 show the simulated and measured DC output voltages and the variation of the RF-to-DC conversion efficiency, respectively, as a function of the effective distance between the RF-EH_2_ and the mobile communication base station (relay antenna).

**Figure 21:**
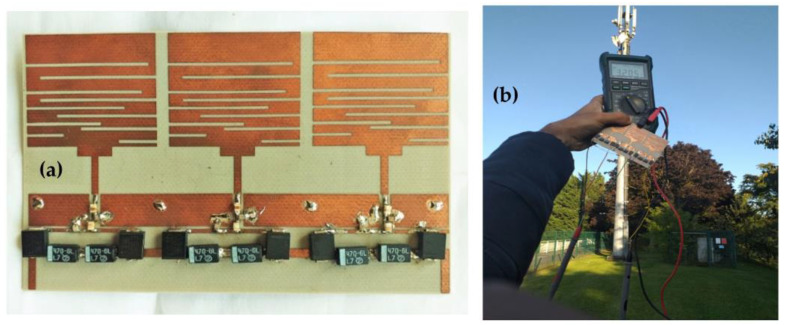
(**a**) Realized RF-EH_2_, (**b**) Prototype of measurement at 22 m from relay antenna.

We note that the output voltage drops rapidly. At 92 m from the base station, the voltage becomes very low 0.2 V and 80 mV at 122 m. Maximum simulated and measured efficiencies of 52% and 50% respectively, are achieved at 45 m from the mobile communication relay antenna. This efficiency decreases quickly beyond 54 m. The DC power supplied by this architecture is 660 μW at the effective distance of 45 m and 3.2 μW at 122 m. The maximum measured DC power of 5.12 mW is achieved at the effective distance of 22 m from the relay antenna. These results demonstrate that this architecture is suitable for height input powers (near relay antenna) and limited for low input powers (far distances from relay antenna). Figure 21 shows the realized RF-EH_2_ with a dimension of 64 × 55 mm^2^.

## 6. The Proposed System (RF-EH)

The RF-EH system consists of switching between the two architectures tested previously by using a switch based on two MOSFET transistors (IRFD_220) in order to realize a suitable system for the low and high input power levels. The switching is done according to the input power levels (close or far from relay antenna). The two outputs, Out_1_ and Out_2_, of the switch are connected to the high and low impedances Z_L1_ and Z_L2_, respectively. 

### 6.1. Design and Realization of Switch Circuit

**Figure 22:**
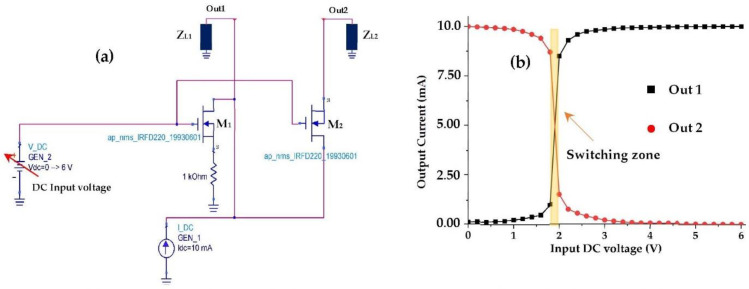
(**a**) Proposed switch circuit, (**b**) Simulated DC output currents (Out1, Out2). Z_L1_ = 7.2 kΩ, Z_L2_ = 0.1 Ω.

Figure 22 depicts the switch circuit with the simulated output currents variation according to the DC input voltage (Gate voltage of the transistors).

**Figure 23:**
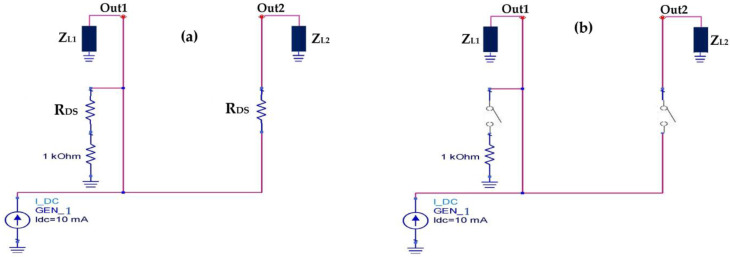
(**a**) Equivalent circuit for high input powers, (**b**) Equivalent circuit for low input powers.

The variable voltage generator simulates the DC voltage supplied by the rectenna (far or near the base station). In the case of high input powers (near the base station), the output voltage is raised, and it is applied at the gate of the transistor. The gate current is null, which implies an infinite input impedance. The voltage delivered by the rectenna is exploited at open load, which makes it possible to reach higher voltages than that of the threshold quickly. In this case, the transistors M_1_ and M_2_ are conducting, which gives the equivalent circuit high input powers illustrated in Figure 23a. For low input powers, both transistors are blocked due to the lower DC voltage provided by the rectenna than the threshold one, which can be modelled by Figure 23b.

For high input powers, both transistors are conducting for a difference of V_G_ − Vs > V_th_, such that the resistance R_DS_ of the transistor IRFD_220 is equal to 0.8 Ω. The majority of the power provided by the source (GEN_1) passes to output 2 with a small ohmic loss due to the R_DS_ resistance. For output 1, the majority of the power supplied by the source (GEN_1) passes through the R_DS_ resistance to the ground with a small leakage to output 1, making this output isolated for high input power. 

For low input powers, both transistors are blocked for a difference of V_G_ − Vs < V_th_ and the R_DS_ resistance is very large (theoretically infinite). In this case, output 2 is completely attenuated and the majority of the power supplied by the source (GEN_1) goes to output 1. Table 6 summarizes the proposed switch states as a function of the input power levels.

The proposed switch is realized on Teflon glass substrate (ε_r_ = 2.1) with 0.67 mm of thickness. The total size of this switch is 15 × 15 mm^2^.

**Figure 24:**
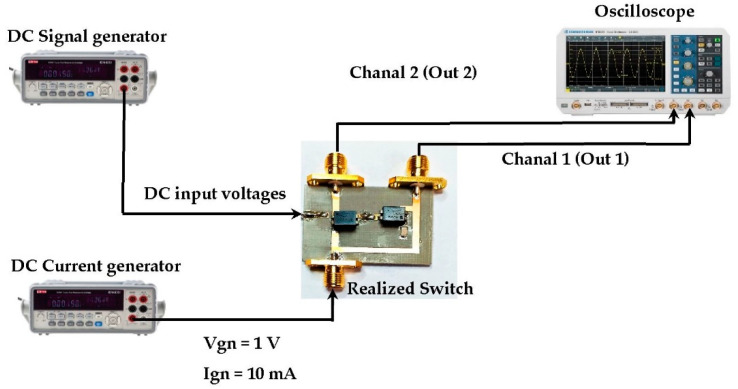
Measurement prototype of the output currents for the first and second outputs of the proposed switch.

**Figure 25:**
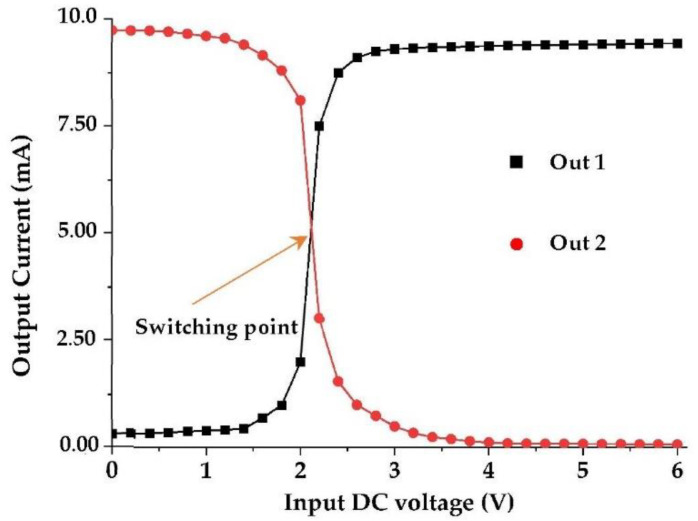
Measured DC output currents (Out1, Out2) of the proposed switch.

Figure 24 and Figure 25 present the realized switch with a measurement prototype and the measured results, respectively.

For I_gn_ = 10 mA and V_gn_ = 1 V, the switching zone is located in the DC input voltage range of [2–2.2] V. The width of the measured switching zone is slightly wider than the simulated one and low leakage currents are observed for both isolated outputs (Out1 and Out2) due to material imperfections. In general, the measurements and simulations are in good agreement. 

The next step is using this switch to realize the proposed RF-EH system, which is discussed in the following paragraph.

### 6.2. Proposed RF-EH System

**Figure 26:**
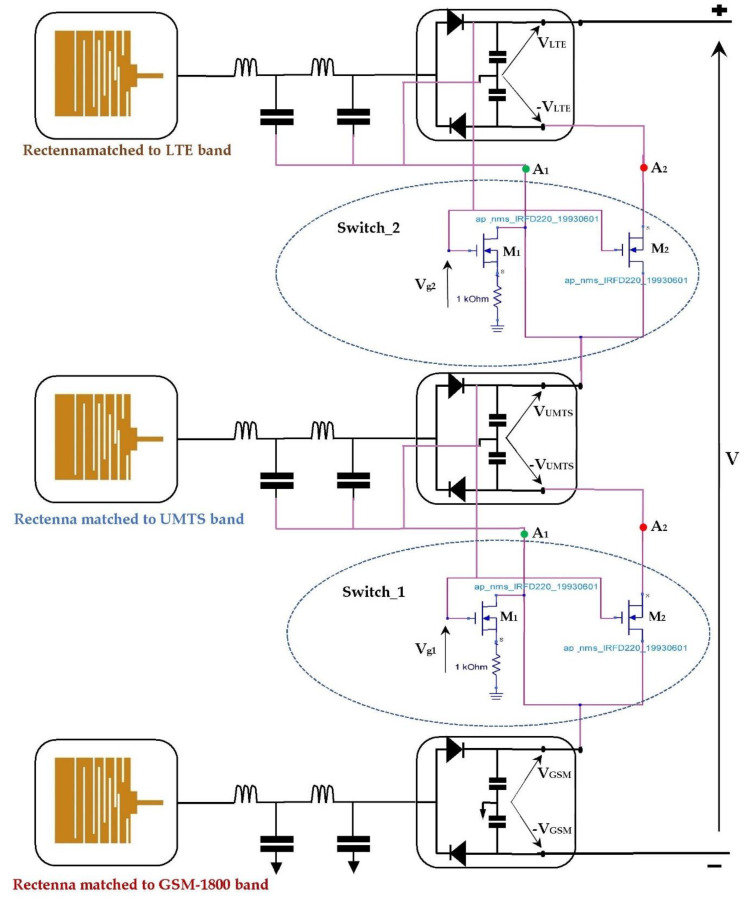
Proposed RF-EH system combining both previous architectures using the studied switch. A_1_ and A_2_ are the two positions corresponding to first and second architectures.

Figure 26 presents the proposed RF-EH system that combines the two architectures studied previously using the presented switch.

**Figure 27:**
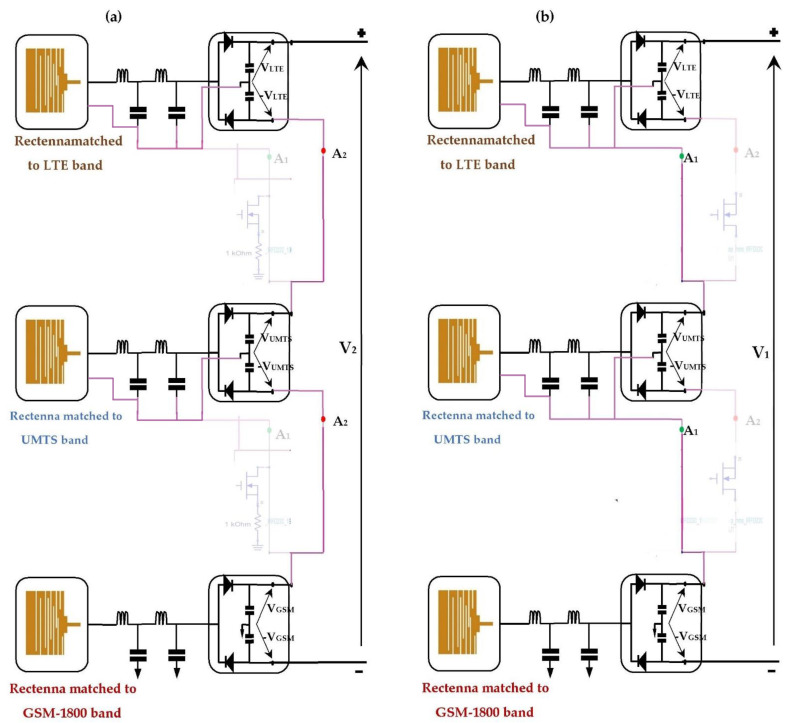
Switching between both previous architectures according to the input power levels. (**a**) for high input power levels, (**b**) for low input power levels.

Due to the infinite gate impedance of the MOSFET, both transistors are controlled by the output voltages of the rectennas for open load (V_g1_ and V_g2_). For high input powers (near relay antenna), the switches are pointing into A_2_. Figure 27a gives the structure of the second architecture. For low input powers (far from relay antenna), the switches pointed into A_1_, resulting in the first architecture, which is presented in Figure 27b.

**Figure 28:**
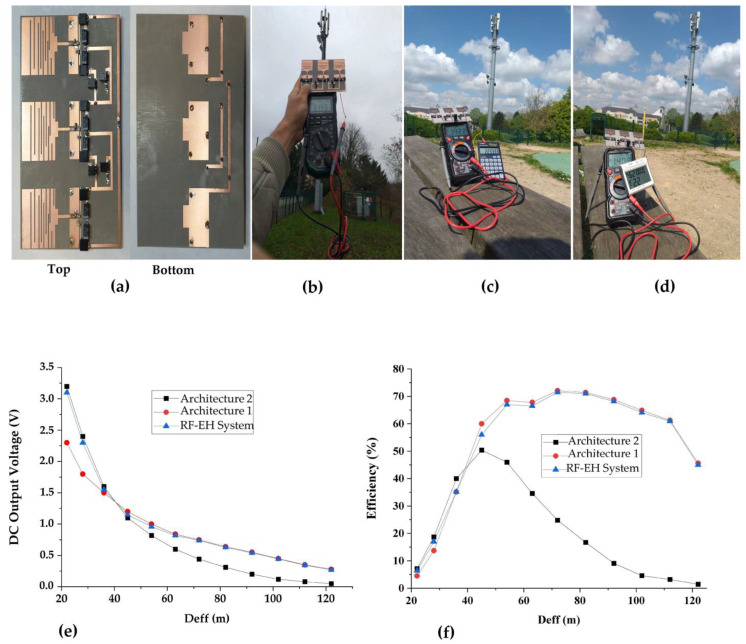
Measurements results: (**a**) realized proposed RF-EH system, (**b**) prototype of measurements, (**c**) Application of the proposed RF-EH system to feed digital calculate at 38 m from relay antenna, (**d**) Application of the proposed RF-EH system to feed temperature sensor at the same distance, (**e**) Measured DC output voltages, and (**f**) Measured RF-to-DC conversion efficiency.

Figure 28 depicts the realized RF-EH system, measurement prototype, measured DC output voltages, and measured RF-to-DC conversion efficiencies for a 2 kΩ resistance load. We can note that the RF-EH system provides a maximum DC voltage of 3.15 V at 22 m from the relay antenna, and a maximum RF-to-DC conversion efficiency of 71.5% at 75 m. The switching from one architecture to another is located at 40 m from the mobile communication base station. From 22 m to 40 m (high input powers), the RF-EH system points to the second architecture, which is more matched for high input power levels. A little difference between the voltages provided by the second architecture (3.2 V) and by the RF-EH system (3.15 V) is due to the transistor leakages for on-transistors (Drain-to-Source resistance R_DS_). From 40 m to 122 m (low input powers), the RF-EH system points to the first architecture, which is more adapted to the low input power levels. Both voltages provided by the RF-EH system and the first architecture are almost the same due to the very low transistor leakages for off-transistors (infinite R_DS_). 

At 122 m, the RF-EH system provides a DC voltage of 0.37 V and RF-to-DC conversion efficiency of 45%.

## 7. Discussion

**Figure 29:**
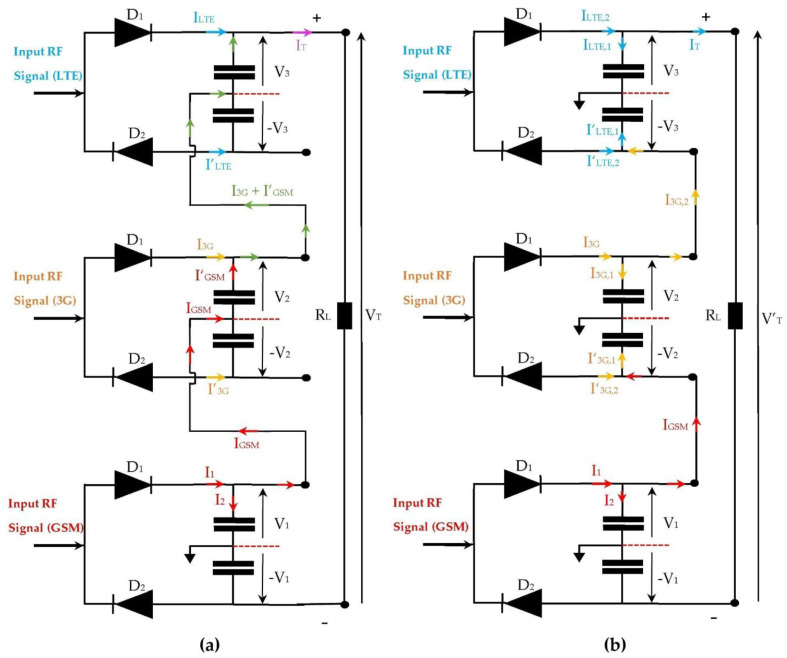
Current flow for both architectures. (**a**) First architecture, (**b**) Second architecture.

The first architecture presented is very suitable for low input power and limited for high input power. This can be explained by the current flow at the link between the rectennas that form each architecture, as shown in Figure 29.

For the first architecture, the link between the rectennas adapted to the GSM and 3G networks is established by the current flow I_GSM_, and the link between the rectennas adapted to the 3G and LTE networks is established by the current flow I_3G_ + I’_GSM_. The RF transmission power of the relay antenna for the 3G network is relatively low compared to that of the GSM network. For low input powers, the current I_3G_ is considerably weakened, and the currents I_GSM_ and I_3G_ + I’_GSM_ ≈ I’_GSM_ remain sufficient to maintain the link between the rectennas up to 122 m from the relay antenna, which guarantees a good matching for low input powers.

For the second architecture, the link between the rectennas matched to the GSM and 3G networks is ensured by the current flow I_GSM_, and the link between the rectennas adapted to the 3G and LTE networks is established by the current flow I_3G,2_, which is a part of the current I_3G_ (I_3G,2_ < I_3G_). For low input power, the current I_3G,2_ is considerably attenuated. The current I_GSM_ is sufficient to maintain the link between the rectennas matched to the GSM and 3G networks up to 122 m from the relay antenna. The current I_3G,2_ is completely attenuated, which prevents the maintenance of the link between the rectennas adapted to the 3G and LTE networks and degrades the performances of this architecture for low input powers.

For high input powers, the second architecture provides higher DC power than the first architecture. For an open load, the DC output voltages supplied by the first and the second architectures are V_T_ = 2V_1_ + V_2_ +V_3_ and V’_T_ = 2(V_1_ +V_2_ +V_3_), respectively. These voltages degrade a little when the R_L_ load is present due to ohmic leakages.

**Figure 30:**
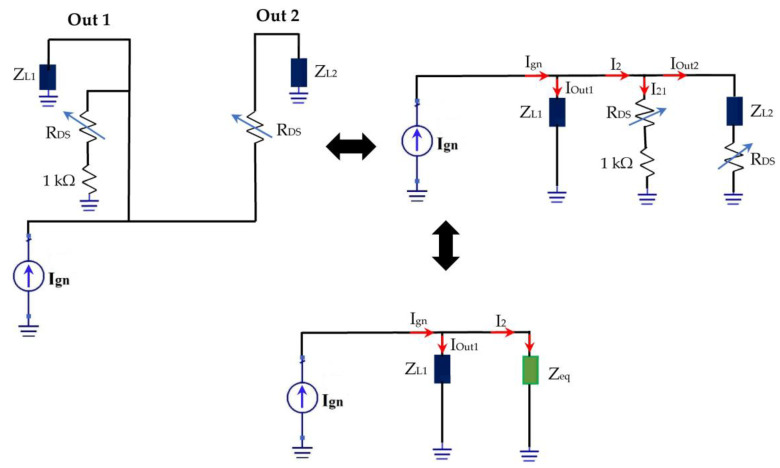
Electrical representation of the proposed switch.

The proposed switch placed between the rectennas in order to select the adapted architecture according to the input power levels is based on two MOSFET transistors. These transistors could be electrically modelized by the source-to-drain equivalent resistor R_DS_, which varies in its function of the input power levels (On/Off Transistor stats) as presented in Figure 30.

(8) $$Z_{\mathrm{eq}}=\frac{(R_{\mathrm{DS}}+1kΩ)\left(Z_{L2}+R_{\mathrm{DS}}\right)}{Z_{L2}+2R_{\mathrm{DS}}+1kΩ}$$

(9) $$I_{\mathrm{Out}1}=\frac{Z_{\mathrm{eq}}}{Z_{L1}+Z_{\mathrm{eq}}}\cdot I_{\mathrm{gn}}$$

(10) $$I_{\mathrm{Out}2}=\frac{Z_{L1}\left(R_{\mathrm{DS}}+1kΩ\right)}{\left(Z_{L2}+2R_{\mathrm{DS}}+1kΩ)\cdot (Z_{L1}+Z_{\mathrm{eq}}\right)}\cdot I_{\mathrm{gn}}$$

For high input power levels, R_DS(on)_ = 0.8 Ω (on-transistor), Z_L1_ = 7.2 kΩ, Z_L2_ = 0.1 Ω, and Z_eq_ = 0.9 Ω.

(11) $$I_{\mathrm{out}1}=1.24\cdot 10^{-4}\cdot I_{\mathrm{gn}}$$

(12) $$I_{\mathrm{out}2}=0.999{\;I}_{\mathrm{gn}}\approx \;I_{\mathrm{gn}}$$

For I_gn_ = 10 mA, I_out1_ = 1.24 µA ≈ 0 A, and I_out2_ = 9.99 mA ≈ I_gn_. It can be noted that the current I_out1_ is almost null (out1 isolated) and that the current I_out2_ is the majority, which implies that the switch points to output 2 for high input powers select the second architecture.

For low input power levels, R_DS(off)_ → ∞ (off-transistor), Z_L1_ = 7.2 kΩ, Z_L2_ = 0.1 Ω, and Z_eq_ → ∞.

(13) $$\begin{array}{c} \lim I_{\mathrm{Out}1}\\ R_{\mathrm{DS}};Z_{\mathrm{eq}}\to \infty \end{array}=I_{\mathrm{gn}}$$

(14) $$\begin{array}{c} \lim I_{\mathrm{Out}2}\\ R_{\mathrm{DS}};Z_{\mathrm{eq}}\to \infty \end{array}=0$$

For I_gn_ = 10 mA, I_out1_ = 10 mA, and I_out2_ = 0 A (in real case I_out2_ is almost null due to leakage currents of the transistor MOSFET). 

We can note that the current I_out2_ is null (out2 isolated) and that the current I_out1_ is the majority, which implies that the switch points to output 1 for low input powers select the first architecture.

In Table 7, a comparison between the proposed RF-EH system and other RF energy harvesting systems reported in the literature is presented.

The proposed system that combines the advantages of both architectures offers a large feeding possibility of many sensors reported in many citations. There are five types of sensors often used: gas sensors, image sensors, pressure sensors, biomedical sensors, and temperature sensors. 

In the following paragraph, we give different sensors cited in the most recent literature according to their DC power consumption.

### 7.1. Gas Sensors

**Figure 31:**
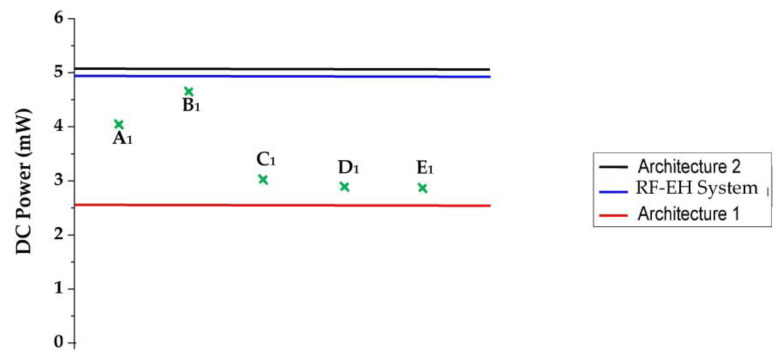
Feeding ability of the system and both architectures at 22 m from relay antenna compared to minimum required power supply of the gas sensors. The DC powers A_1_, B_1_, C_1_, D_1_ and E_1_ correspond to the power supply of sensors given in the references [26,27,28,29,30], respectively.

In [26], the authors propose a Si FET-type gas sensor with a localized micro-heater capable of heating up to 124 °C with a power consumption of 4 mW. In [27], a high linearity detection circuit with a constant detection voltage for a resistive gas sensor is proposed. The maximum power consumption of this circuit is only 4.6 mW. The authors of [28] report a hexagonal gas sensor cell for multi-channel gas detection. Six sensors are integrated with a hexagonal micro-hotplate to make up the sensor cell. The average power consumption of the sensor cell is 3.03 mW when the sensor cell starts working. In [29], a novel metal oxide semiconductor (MOS) gas sensor based on a single cantilever is reported. The static power consumption is 2.96 mW. The authors of [30] propose the hydrogen gas detection property of Pt-AlGaN/GaN high electron mobility transistor (HEMT) sensors with a recessed gate structure. The power consumption of the sensor is 2.95 mW. Figure 31 presents the feeding abilities of both proposed architectures and the system compared to the minimum required power supply of these sensors.

As we can see, the RF-EH system as well as the second architecture could feed the cited sensors [26,27,28,29,30] at 22 m from relay antenna. Contrary to the first architecture, which provides a DC power lower than what theses sensors require to be turned on given that they are far from relay antenna (D > 30 m), so these sensors couldn’t be powered.

### 7.2. Image Sensors

The authors of [31] propose a single-pixel sensor for near-field imaging based on the startup time of an oscillator. The average power consumption of this sensor is 2.7 mW. In [32], the authors report a counter structure for a single slope analog-to-digital converter that is parallel to a column (SS-ADC) in CMOS image sensors. The total power consumption is 2.25 mW for a 640 × 480 effective image resolution at 60 frame rates. In [33], the authors propose a mixed-signal perception chip, in which a 32 × 32 ADC-free image sensor and a BNN processing array are directly integrated into a standard 180 nm CMOS process. The entire processing system consumes only 1.8 mW. In [34], the authors report a CMOS image sensor (CIS) with column-parallel single-shot compressive sensing (CS) for always-on Internet-of-Things (IoT) application. A prototype VGA image sensor consumes only 0.7 mW at 45 frames/s. In [35], a CMOS image sensor with a programmable kernel for feature extraction is proposed. The power consumption of this sensor is 117 μW for 480 frames/s. 

**Figure 32:**
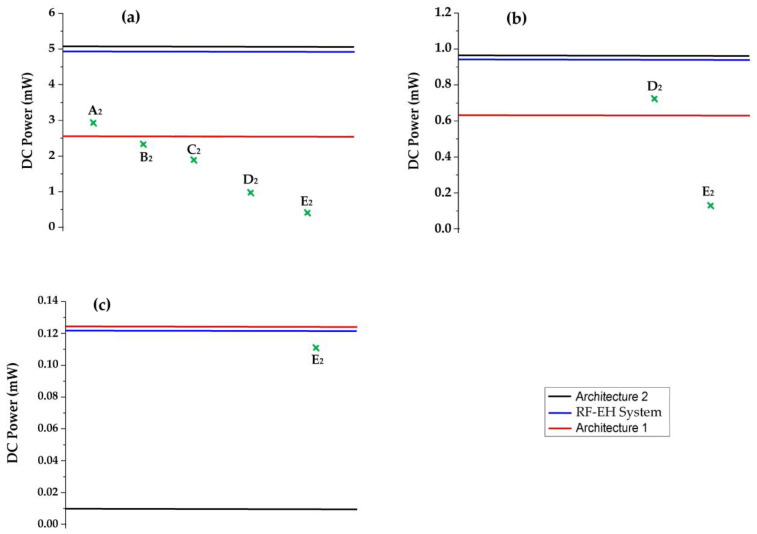
Feeding ability of the RF-EH system and architectures at (**a**): 22 m, (**b**): 40 m, and (**c**): 110 m from relay antenna compared to minimum required power supply of the image sensors. The DC powers A_2_, B_2_, C_2_, D_2_ and E_2_ correspond to the power supply of sensors proposed in the references [31,32,33,34,35], respectively.

Figure 32 depicts the minimum required power supply of these sensors and the DC output power supplied by both architectures and the RF-EH system at different distances from the relay antenna.

We can note that the RF-EH system and the second architecture could feed all sensors cited in [31,32,33] at 22 m form the relay antenna. The first architecture provides enough DC power to feed the sensors of [32,33], except for that of [31]. At 40 m, the proposed RF-EH system and the second architecture could feed the sensors of [34,35]. The first architecture supplies a sufficient DC power to feed only the sensor reported in [35]. At 110 m from the relay antenna, the second architecture provides insufficient DC power to feed any sensor. The proposed RF-EH system and the first architecture could feed the sensor cited in [35]. 

### 7.3. Pressure Sensors

In [36], a pressure sensor array with a low-power near-sensor CMOS chip for Human Gait Monitoring is proposed. The average power consumption of this sensor is 1.45 mW. The authors of [37] report an iontronic pressure sensor based on organic electrochemical transistors. The average power consumption of this sensor is 0.5 mW. In [38], a thin-film MOSFET-based pressure sensor is proposed. The power consumption of this sensor is 390 μW. In [39], the authors propose a sensor to detect pressure changes from 30 mmHg to 1000 mHg. It consumes a power of 72 μW.

**Figure 33:**
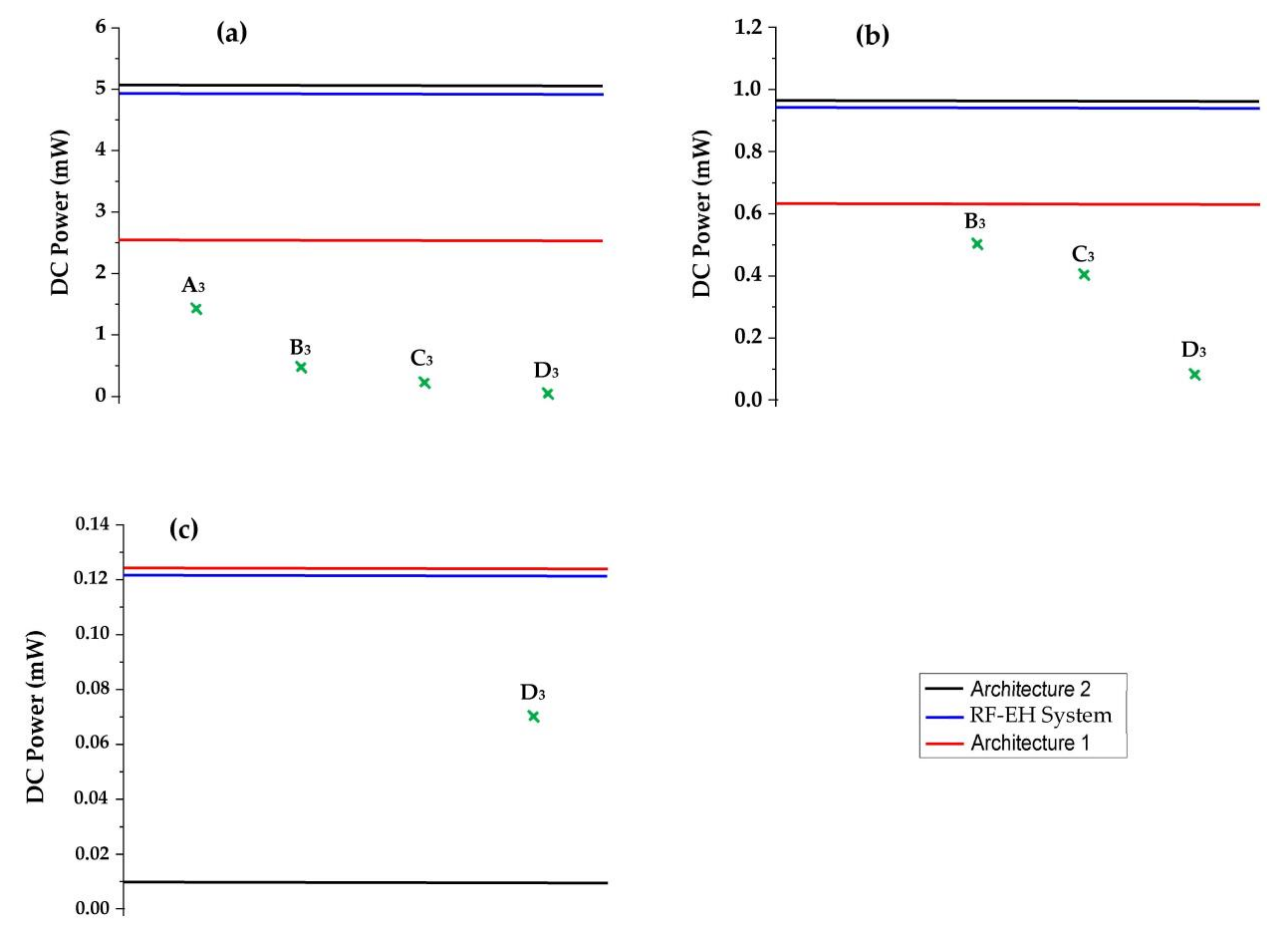
Feeding ability of the RF-EH system and architectures at (**a**): 22 m, (**b**): 40 m, and (**c**): 110 m from relay antenna compared to minimum required power supply of the pressure sensors. The DC powers A_3_, B_3_, C_3_ and D_3_ correspond to the power supply of sensors cited in the references [36,37,38,39], respectively.

Figure 33 shows the feeding abilities of the RF-EH system and both architectures according to the distance from relay antenna.

As we can see, at 22 m from the relay antenna the RF-EH system, as well as both architectures, could feed all pressure sensors cited in [36,37,38,39]. At 40 m, the sensors reported in [37,38,39] could be fed. At 110 m, the RF-EH system and the first architecture could feed the sensor from [39]. The second architecture doesn’t provide enough DC power.

### 7.4. Biomedical Sensors

**Figure 34:**
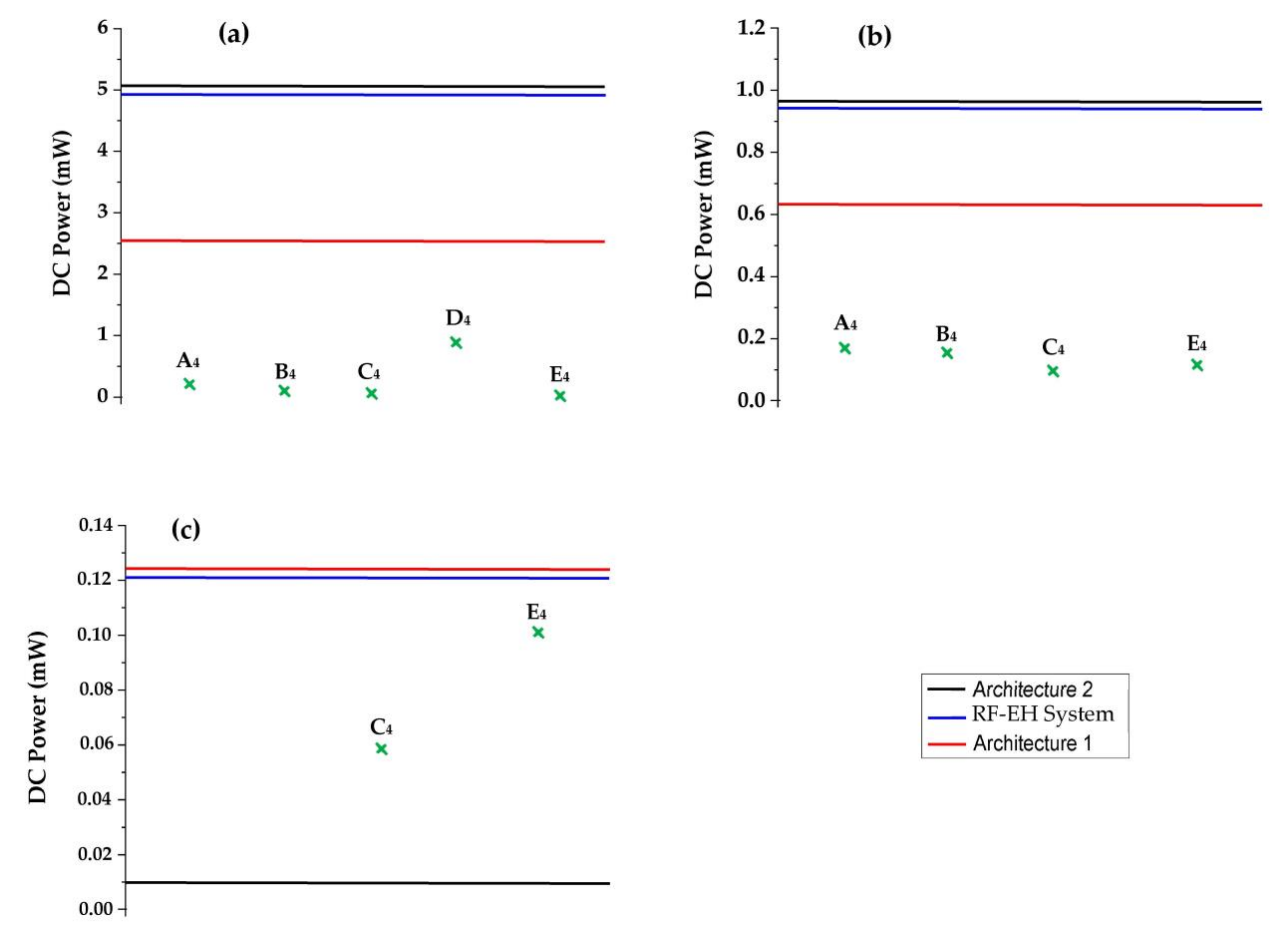
Feeding ability of the RF-EH system and architectures at (**a**): 22 m, (**b**): 40 m, and (**c**): 110 m from relay antenna compared to minimum required power supply of the biomedical sensors. The DC powers A_4_, B_4_, C_4_, D_4_ and E_4_ correspond to the power supply of sensors presented in the references [40,41,42,43,44], respectively.

The proposed system offers a large feeding possibility for many sensors reported in many citations. In [40], the authors propose a Photoplethysmography (PPG) sensory system for continuous health monitoring. The average power consumption of this sensor is 196 μW, including the Reference/Bias generation and analog-to-digital conversion section ADC. The same sensor topology is presented in [41] and [42] with a reduced power consumption level of 121 μW and 50.75 μW, respectively. The sensor proposed in [43] is a CMOS Reconfigurable Multi-Sensor SoC for Biomedical Applications. It consists of three blocks that consume the most energy (10-bit SAR-ADC block, OOK transmitter, Digital processor, and bias circuit and buffer). The total power consumed by this sensor is 942.9 μW. In [44], The authors propose an optical Sensor for Accurate Heartbeat Monitoring with a voltage range of [1.8–3.3] V for 60 μA, which gives a power consumption range of [108–198] μW. Figure 34 shows the feeding ability of both architectures and the RF-EH system in the function of the distance from the relay antenna.

We can remark that all sensors could be powered by the RF-EH system and both architectures at 22 m from the relay antenna. At 40 m, the RF-EH system and the second architecture allow for all sensors feeding. The first architecture provides an insufficient DC power to feed the sensor reported in [43]. At 110 m, the RF-EH system and the first architecture could feed only the sensors from [42,44]. The second architecture couldn’t feed any sensor at this distance.

### 7.5. Temperature Sensors

**Figure 35:**
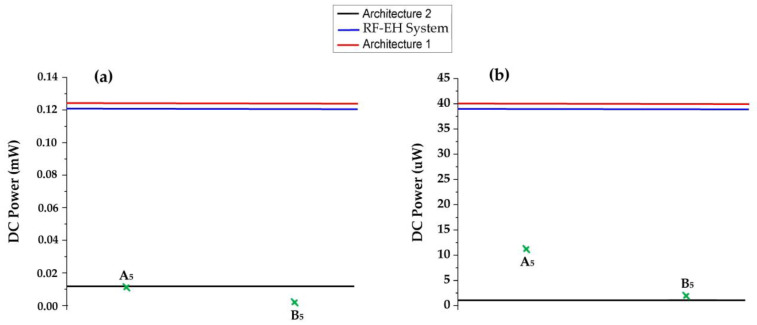
Feeding ability of the proposed system and architectures at (**a**): 110 m, (**b**): 122 m from relay antenna compared to minimum required power supply of the temperature sensors. The DC powers A_5_ and B_5_ correspond to the power supply of sensors reported in the references [45,46], respectively.

In [45], the authors report a spintronic/CMOS-based thermal sensors. This consists of a thermal aware system composed of hundreds of distributed thermal sensor nodes. This sensor system consumes a power of 11.9 μW during its on-state. In [46], an integrated pulse width modulated (PWM) temperature sensor is proposed. The power consumption of this sensor is 47.2 nW (for 27 °C) and 17.6 nW (for −20 °C). Both sensors could be fed by the RF-EH system as well as the two architectures within the distance range of [22–110] m from the relay antenna, as presented in Figure 35. At 122 m, the second architecture provides an almost null DC power that doesn’t allow for the feeding of these sensors. 

## 8. Application of RF-EH System to Feed WSN

### 8.1. Ground Effect on RF Energy Harvesting Process

The presence of an obstacle, especially when it is near the radiating element, can change a little of the overall radiation properties of the RF-EH antenna. In practice, the most common obstacle that is always present is the ground. Any energy provided from the radiating element (patch) directed towards the ground will be reflected. The amount of reflected energy and its direction are controlled by the geometry and the constituent parameters of the ground. 

In general, the ground is a lossy medium (σ ≠ 0) whose effective conductivity increases with frequency. Thus, it acts as a very good conductor for certain frequencies, depending mainly on its composition and moisture content. The Volumetric Water Content (VWC) parameter is the main factor affecting the response of an antenna. Thus, it broadens the bandwidth and modifies the radiation pattern by increasing the directivity and gain [47,48,49].

In [47,48], the presence of the ground (VWC) allows a bandwidth broadening from 8 GHz to a band of 10.5 GHz. In [49], the presence of the ground provides an advantage to the antenna by expanding the bandwidth from 3 GHz to 3.8 GHz. In our case, the energy harvesting process will not be affected by the presence of the ground because even if the bandwidth of the antenna is widened, the rectifiers remain suitable for the GSM-1800, UMTS, and LTE bands. A slight improvement in gain [50] would improve the received RF power, and therefore the DC output power.

### 8.2. Presentation of WSN System to Be Powered by the Proposed RF-EH System

Many applications of wireless sensor networks are limited by the insufficient battery life of the sensors. The power consumption of processors and microcontrollers is being significantly reduced due to new advances in micro-electronics. This gives the possibility for using RF energy harvesting sources to feed wireless sensor nodes [51].

Wireless sensor systems are designed mainly from several sensor nodes that are dispersed around several management stations called cluster heads (CH), which form clusters. These are located around a sink station. Each sensor node collects information from its environment and transmits it to its cluster head (CH), then each CH communicates with its sink station via wireless communication that allows users to access this information.

**Figure 36:**
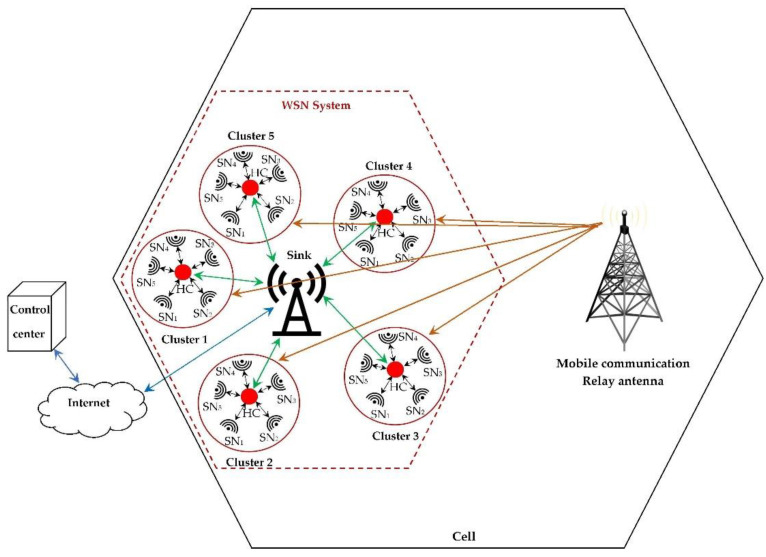
WSN system architecture within mobile communication cell.

These sensor systems are deployed inside the cells of the mobile communication network managed by relay antennas as shown in Figure 36 [52,53].

The proposed RF-EH system mainly exploits the electromagnetic waves radiated by these relay antennas due to their high transmission power and the multitude of bands available to feed each sensor node. The RF-EH system can also exploit the electromagnetic waves coming from the sink stations as well as from the sensors themselves.

To properly study how the proposed RF-EH system self-powers its wireless sensor systems (RF-EHWSN), two cases can be discussed.

- WSN system close to the relay antenna.
- WSN system far from the relay antenna.

### 8.3. WSN System Close to the Relay Antenna

In this case, the distance between the relay antenna and the RF-EHWSN is small, which implies a high RF power received by the RF-EH system. This allows it to provide a high enough DC power to feed the sensor nodes. The output DC power could be exploited directly by using a DC voltage regulator connected between the output of the RF-EH system and the sensor feed point.

### 8.4. WSN System Far from the Relay Antenna

In this case, the distance between the relay antenna and the RF-EHWSNs is large, which greatly reduces the RF power received by the RF-EH system, and thus the output DC power. For this scenario, the direct use of the output DC power does not ensure a good self-powering of the sensor nodes. A super-capacitor placed between the RF-EH system and the sensors is recommended. This super-capacitor allows for the storage of the energy supplied by the RF-EH system while the sensors are in standby mode. The full charge of these super-capacitors takes a relatively large amount of time, which could impact the communication process between the sensor nodes. The addition of a power management block is essential to solve this problem. The power management circuit consists of a DC–DC converter for optimizing the energy transfer from the output of the RF-EH system to the accumulator, and a linear low-dropout (LDO) voltage regulator for generating a suitable sensor supply voltage [54].

### 8.5. WSN System Self-Powering

**Figure 37:**
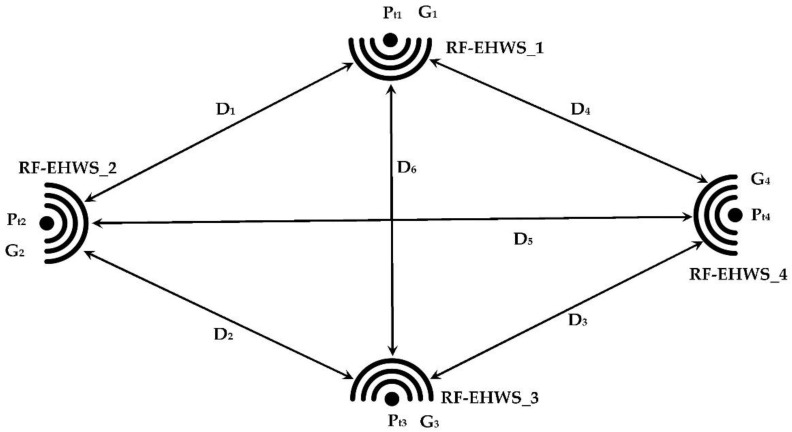
WSN system architecture for self-powering process.

The exploitation of RF waves during an information exchange between sensor nodes could be used to self-power them. Indeed, in the case where the ZigBee protocol was adopted by the WSN system, for example, each RF-EHWS (RF energy harvesting of Wireless Sensor) retrieves the electromagnetic waves coming from the other transmitting sensors. The process is similar for all sensor nodes that exist in the WSN system, as shown in Figure 37.

*P_ti_*, *G_i_*, and *D_i_* are the transmission power of each sensor, gain of sensor antenna, and the distances between sensors, respectively.

The received power by each RF-EHWS could be expressed by the following equations using the Friis formula:

(15) $$P_{r}=G_{t}G_{r}{\left(\frac{\lambda }{4\pi D}\right)}^{2}P_{t}$$

where *P_r_, G_t_, G_r_, P_t_, D*, and λ are the transmission power, transmission gain, reception gain, the distance between sensors, and the wavelength, respectively.

RF-EHWSN_1 receives power from the three other sensors, which gives Equations (16)–(19):

(16) $$P_{r1}=G_{t2}G_{r1}{\left(\frac{\lambda }{4\pi D_{1}}\right)}^{2}P_{t2}+G_{t3}G_{r1}{\left(\frac{\lambda }{4\pi D_{6}}\right)}^{2}P_{t3}+G_{t4}G_{r1}{\left(\frac{\lambda }{4\pi D_{4}}\right)}^{2}P_{t4}$$

(17) $$P_{r2}=G_{t1}G_{r1}{\left(\frac{\lambda }{4\pi D_{1}}\right)}^{2}P_{t1}+G_{t3}G_{r1}{\left(\frac{\lambda }{4\pi D_{2}}\right)}^{2}P_{t3}+G_{t4}G_{r1}{\left(\frac{\lambda }{4\pi D_{5}}\right)}^{2}P_{t4}$$

(18) $$P_{r3}=G_{t1}G_{r1}{\left(\frac{\lambda }{4\pi D_{6}}\right)}^{2}P_{t1}+G_{t2}G_{r1}{\left(\frac{\lambda }{4\pi D_{2}}\right)}^{2}P_{t2}+G_{t4}G_{r1}{\left(\frac{\lambda }{4\pi D_{3}}\right)}^{2}P_{t4}$$

(19) $$P_{r4}=G_{t1}G_{r1}{\left(\frac{\lambda }{4\pi D_{4}}\right)}^{2}P_{t1}+G_{t2}G_{r1}{\left(\frac{\lambda }{4\pi D_{5}}\right)}^{2}P_{t2}+G_{t3}G_{r1}{\left(\frac{\lambda }{4\pi D_{3}}\right)}^{2}P_{t3}$$

where *P_ri_* are the received RF powers by RF-EHWS_i.

As we can see, each RF-RHWS receives three powers, this increases the output DC voltages provided by the RF-EH systems, which allows for the self-powering of WSNs. In order to use the proposed RF-EH system efficiently, it is necessary to match all the rectennas of the system to the communication frequency of the WSN.

### 8.6. Obstacle Facing the Application of RF Energy Harvesting in WSNs and Proposed Protocols

RF energy is abundantly available in the environment from various sources of electromagnetic radiation, which allows it to be used with WSNs. This type of energy effectively provides mobile self-power, which is consistent with the wireless functionality of the WSN. 

**Figure 38:**
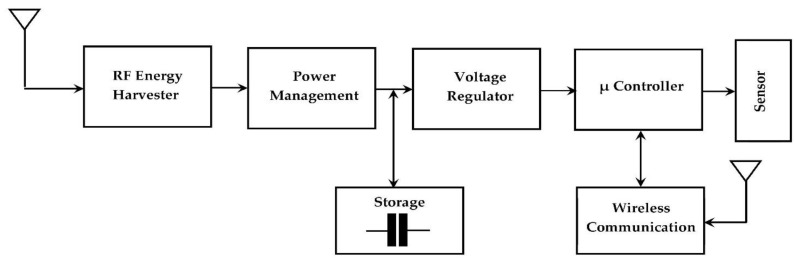
RF-EHWSN system complete architecture.

The real challenge facing the application of RF energy harvesting in WSNs is the difficulty of dividing the sensor operating time efficiently between the charging process and data transmission times. For this purpose, several protocols have been proposed in the literature to solve this problem [55,56]. These protocols are applied to a microcontroller placed between the storage capacitor and the node sensor, as presented in Figure 38 [57].

Two MAC (Medium Access Control) protocols take into account the RF energy harvesting applications: RF-AASP (Radio Frequency energy harvesting process based on active and adaptive sleep period) and RF-MAC (RF Medium Access Control). The RF-AASP determines the charging time of the super-capacitor based on the data traffic and the RF-MAC determines the charging time based on the sensor’s participation in the data communication.

In [55], an RF-AASP algorithm is proposed to adaptively manage the sleep period of a sensor. The period varies according to the traffic model and the available energy of the sensors. A low-energy sensor checks the traffic model and the quality of service (QoS) satisfaction. Based on these results, the sensor adjusts parameters like Beacon Order (BO) and Super frame Order (SO). The two adjusted parameters determine the sleep period by the equation t_sleep_ = 2BO − 2SO. The sensor harvests energy during the sleep period to charge the supercapacitors. In [56], an RF-MAC algorithm is proposed with a new approach for RF energy harvesting. The energy harvesting is done with the RFE (Request-for-Energy) packet. A low energy sensor dispatches the RFE packet. Peripheral energy transmitters (for this protocol it is the Sink station) respond with CFE (Cleared-for-Energy) packets. When the sensor dispatches the ACK, the sink station emits energy. In this case, the charging time depends on the value of the Important Index (IDX), which indicates how the sensor participated in the data communication on the channel. This algorithm ensures continuous data communication when there is no energy demand. However, when there is a frequent demand for energy, starvation can occur. A sensor that sends data falls into starvation because of the higher priority of the energy demand.

## 9. Conclusions

In this paper, a new RF energy harvesting system is proposed in order to enhance the DC output voltage for low power WSN feeding. It is based on two architectures, one suitable for low input powers and the other for high input powers. Input power-controlled switching circuits are used to select the more suitable architecture. Both architectures and the system have been designed and realized on Teflon glass hybrid technology and experimentally characterized separately. The efficiency variation as a function of the load resistance yield were at a load of 2 kΩ for maximum efficiency. This value is used for all measurements. DC output voltages and efficiencies have been measured as a function of the distance from the mobile communication base station (relay antenna). The transfer function of the switches has been studied, simulated, and experimentally characterized in order to verify their functionality. The first architecture supplies a maximum DC output voltage of 2.3 V at 22 m from the base station and presents a maximum efficiency of 72% at 70 m. The second one provides a maximum DC output voltage of 3.2 V at 22 m from the base station and a maximum efficiency of 50% at 40 m. The proposed system provides a maximum DC output voltage of 3.15 V at 22 m from the relay antenna and a maximum efficiency of 71.5% at 70 m from the relay antenna. A DC voltage of 0.37 V is provided by the proposed RF-EH system at 122 m from the relay antenna with an RF-to-DC conversion efficiency of 43%. 

Each rectenna that forms both architectures and the RF-EH system consists of a new multi-band antenna matched to a rectifier. The antenna covers all the mobile communications and wireless network bands (GSM-1800, UMTS, LTE-2.3, Wi-Fi, and LTE-2.6). The antenna has been characterized by simulating and measuring its S_11_ parameter and radiation pattern. The measured efficiencies of the antenna are 70%, 65%, and 68.5% for 1.8 GHz, 2.1 GHz, and 2.66 GHz, respectively. 

Finally, the functioning of the architectures as well as that of the switching circuits has been explained and discussed. The DC output voltages provided by the architectures and by the RF-EH system have been applied to several types of sensors reported in the literature in order to ensure the operation of these sensors as a function of the distance from the relay antenna.

## Figures and Tables

**Table 1:** Measured received powers as a function of distance from the mobile communication antenna relay for the three selected networks (GSM-1800, UMTS-2100 and LTE-2.6) in mW.

D_eff_ (m)	22	28	36	45	54	63	72	82	92	102	112	122
P(GSM)	28	7.2	1.8	0.6	0.35	0.25	0.17	0.12	0.09	0.07	0.05	0.043
P(UMTS)	18	2.8	0.8	0.35	0.22	0.17	0.13	0.097	0.08	0.05	0.032	0.028
P(LTE)	12.8	1.8	0.6	0.25	0.16	0.1	0.08	0.07	0.05	0.036	0.018	0.015

**Table 2:** Substrates used in the design of the RF-EH systems.

Ref.	[10]	[11]	[12]	[13]	[14]	[15]	[16]	[17]	[18]	[19]	[20]	[21]	[22]	[23]	[24]
**Substrates**	RT/Duroid 6002	Flet Polyester/Woven Polyester	FR-4	Rogers 5880	Rogers 5880	FR-4	Roger 4003C	Rogers RT6002	Rogers 5880	Rogers 3210	FR-4	Rogers 4003C	FR-4	Rogers 4003C	Rogers 5880
**ε_r_**	2.94	1.2/1.5	4.4	2.2	2.2	4.4	3.55	2.94	2.2	10.2	4.4	3.38	4.4	3.38	2.2

**Table 3:** All antenna dimensions.

Dimensions	Values (mm)
L_t_	40
W_t_	30
W	2
L_a_	3.5
L_e_	13.5
L_1_	6
L_2_	6
L_3_	3
L_4_	3.5
L_5_	2
L_6_	2.5
M_1_	1.2
M_2_	2
M_3_	3
M_4_	2
M_5_	7
M_6_	6
M_7_	13
M_8_	12
M_9_	8
W_1_	9
W_2_	9.5
W_g_	5
L_g_	9

**Table 4:** Electrical ideal element values of the impedance matching circuits for different frequency bands.

Frequency	L_1_ (nH)	L_2_ (nH)	C_1_ (pF)	C_2_ (pF)
1.8 GHz	1.5	7.9	7.5	0.55
2.11 GHz	1.2	3.9	9.9	1.1
2.66 GHz	0.2	2.7	6.5	0.8

**Table 5:** Values of the parasitic elements of the used inductors and capacitors for the three frequency bands.

Frequency	L_s1_ (pH)	L_s2_ (pH)	C_L1_ (pF)	C_L2_ (pF)	R_LS1_ (mΩ)	R_LS2_ (mΩ)	R_S1_ (mΩ)	R_s2_ (mΩ)
1.8 GHz	0.25	7.9	1.8	0.55	60	52	96	82
2.11 GHz	0.17	5	1.98	0.6	78	50	80	70
2.66 GHz	0.1	0.1	14.8	0.5	150	150	5	50

**Table 6:** Output stats of the proposed switch according to the input power levels.

Input Power Levels	Low Input Power	High Input Power
Output 1	ON	OFF
Output 2	OFF	ON

**Table 7:** Comparison between proposed RF-EH system performances and other systems reported in the literature.

References	Input Power (dBm)	Freq. Band (GHz)	Max RF-DC Eff (%)	Maximum Output Voltage (V)	System Dimensions (mm^3^)	Minimum Load (kΩ)
[10]	0	2.45	81	1 V at −3.5 dBm	125 × 80 × 0.25	1.4
[11]	−7	2.45	28.7	3 V at −7 dBm	500 × 64 × 3.3	4
[12]	−8	0.823–1.337	61.7	2 V at 0 dBm	120 × 120 × 0.52	10
[13]	3	0.9–3	73.4	1.2 V at 3 dBm	200 × 180 × 0.787	1
[14]	−1	0.45–0.9	75	1 V at −8 dBm	160 × 160 × 1.5	15
[15]	−10	0.91–2.55	68	0.243 V at −10 dBm	165 × 125 × 0.8	4.7
[16]	−6	1.4–2.95	27.5	0.484 V at −6 dBm	100 × 80 × 0.87	0.8
[17]	−3	1.7–1.8 and 2.1–2.7	67	1.1 V at −3 dBm	145 × 145 × 1.53	2
[18]	−5	0.866, 0.925 and 2.45	65	0.7 V Ambient	115 × 71 × 0.787	10
[19]	0	2.45 and 5.8	58	NA	64 × 64 × 1.24	0.7
[20]	−27	0.95, 1.74, 2.12, 2.42 and 2.90	66.5	0.797 V Ambient	220 × 220 × 30	2.11
[21]	−10	1.8	51.3	0.129 V Ambient	100 × 100 × 60	6
[22]	−3	2.45	45.3	0.782 V Ambient	337 × 226 × 10	3
[23]	10	5.8	68	1.1 V at 10 dBm	86 × 24 × 0.813	0.22
[24]	6.5	5.2 and 5.8	54.9	0.7 V at 6.5 dBm	87 × 90.9 × 0.787	0.2
[25]	5.8	0.9, 1.8, 2.1 and 2.45	84	0.9 V at −15 dBm	100 × 100 × NA	11
**This Work**	**10**	**1.8, 2.1 and 2.66**	**71.5%**	**3.1 V Ambient**	**65 × 64 × 0.67**	**2**

## Data Availability

Not applicable.

## References

[1] Zhang M., Liu X., Guo H., Yang X., Xie D. Development of high-efficiency rectification circuit for RF energy harvesting. Proceedings of the 2015 Asia-Pacific Microwave Conference (APMC).

[2] Saraiva H.M., Borges L.M., Pinho P., Goncalves R., Chavez-Santiago R., Barroca N., Tavares J., Gouveia P.T., Carvalho N.B., Balasingham I. Experimental Characterization of Wearable Antennas and Circuits for RF Energy Harvesting in WBANs. Proceedings of the 2014 IEEE 79th Vehicular Technology Conference (VTC Spring).

[3] Chaour I., Fakhfakh A., Kanoun O. (2017). Enhanced Passive RF-DC Converter Circuit Efficiency for Low RF Energy Harvesting. Sensors.

[4] Taghadosi M., Albasha L., Quadir N.A., Rahama Y.A., Qaddoumi N. (2017). High Efficiency Energy Harvesters in 65nm CMOS Process for Autonomous IoT Sensor Applications. IEEE Access.

[5] Zhang L., Cheng X., Deng X. (2019). A modified Dickson’s charge pump circuit with high output voltage and high pumping efficiency. Analog Integr. Circuits Signal Process..

[6] Okba A., Takacs A., Aubert H., Charlot S., Calmon P.F. (2017). Multiband rectenna for microwave applications. Comptes Rendus. Phys..

[7] Tung N. Multi-band ambient RF energy harvesting rectifier for autonomous Wireless Sensor networks. Proceedings of the 2016 IEEE Region 10 Conference (TENCON).

[8] Nimo A., Beckedahl T., Ostertag T., Reindl L. (2015). Analysis of Passive RF-DC Power Rectification and Harvesting Wireless RF Energy for Micro watt Sensors. AIMS Energy.

[9] Singh N., Kanaujia B.K., Beg M.T., Mainuddin, Khan T., Kumar S. (2018). A dual polarized multiband rectenna for RF energy harvesting. AEU-Int. J. Electron. Commun..

[10] Sun H., Guo Y., He M., Zhong Z. (2012). Design of a High-Efficiency 2.45-GHz Rectenna for Low-Input-Power Energy Harvesting. IEEE Antennas Wirel. Propag. Lett..

[11] Adami S.-E., Proynov P., Hilton G.S., Yang G., Zhang C., Zhu D., Li Y., Beeby S.P., Craddock I.J., Stark B.H. (2018). A Flexible 2.45-GHz Power Harvesting Wristband with Net System Output From −24.3 dBm of RF Power. IEEE Trans. Microw. Theory Tech..

[12] Vishal S., Shankar P.V., Vivek S. (2021). A Wideband Star-Shaped Rectenna for RF Energy Harvesting in GSM Band. Advances in Electromechanical Technologies.

[13] Moreno J.M., Vázquez A.S.M., Barragán C.A.B., González J.M.V., Rosas J.C.A. (2020). Radio Frequency Energy Harvesting System Making Use of 180° Hybrid Couplers and Multiple Antennas to Improve the DC Output Voltage. IEEE Lat. Am. Trans..

[14] Song C., Huang Y., Zhou J., Carter P. (2017). Improved Ultrawideband Rectennas Using Hybrid Resistance Compression Technique. IEEE Trans. Antennas Propag..

[15] Fakharian M.M. (2020). A Wideband Rectenna Using High Gain Fractal Planar Monopole Antenna Array for RF Energy Scavenging. Int. J. Antennas Propag..

[16] Nguyen H.Q., Le M.T. (2021). Multiband Ambient RF Energy Harvester with High Gain Wideband Circularly Polarized Antenna toward Self-Powered Wireless Sensors. Sensors.

[17] Song C., Lu P., Shen S. (2021). Highly Efficient Omnidirectional Integrated Multiband Wireless Energy Harvesters for Compact Sensor Nodes of Internet-of-Things. IEEE Trans. Ind. Electron..

[18] Quddious A., Zahid S., Tahir F.A., Antoniades M.A., Vryonides P., Nikolaou S. (2021). Dual-Band Compact Rectenna for UHF and ISM Wireless Power Transfer Systems. IEEE Trans. Antennas Propag..

[19] Li L., Zhang X., Song C., Zhang W., Jia T., Huang Y. (2021). Compact Dual-Band, Wide-Angle, Polarization- Angle -Independent Rectifying Metasurface for Ambient Energy Harvesting and Wireless Power Transfer. IEEE Trans. Microw. Theory Tech..

[20] Roy S., Tiang R.J.-J., Roslee M.B., Ahmed M.T., Mahmud M.A.P. (2021). Quad-Band Multiport Rectenna for RF Energy Harvesting. IEEE Access.

[21] Shen S., Zhang Y., Chiu C.-Y., Murch R. (2021). Directional Multiport Ambient RF Energy-Harvesting System for the Internet of Things. IEEE Internet Things J..

[22] Hu Y.-Y., Sun S., Xu H., Sun H. (2019). Grid-Array Rectenna with Wide Angle Coverage for Effectively Harvesting RF Energy of Low Power Density. IEEE Trans. Microw. Theory Tech..

[23] Lu P., Song C., Cheng F., Zhang B., Huang K. (2020). A Self-Biased Adaptive Reconfigurable Rectenna for Microwave Power Transmission. IEEE Trans. Power Electron..

[24] Lu P., Huang K.M., Yang Y., Cheng F., Wu L. (2019). Frequency-Reconfigurable Rectenna with an Adaptive Matching Stub for Microwave Power Transmission. IEEE Antennas Wirel. Propag. Lett..

[25] Kuhn V., Lahuec C., Seguin F., Person C. (2015). A Multi-Band Stacked RF Energy Harvester with RF-to-DC Efficiency Up to 84%. IEEE Trans. Microw. Theory Tech..

[26] Jang D., Jung G., Jeong Y., Hong Y., Hong S., Shin W., Chang K.S., Jeong C.B., Park B.-G., Lee J.-H. Efficient Integration of Si FET-type Gas Sensors and Barometric Pressure Sensors on the Same Substrate. Proceedings of the 2019 IEEE International Electron Devices Meeting (IEDM).

[27] Li C., Chen M., Peng S., Qi W., Wang N., Rong Q., Chen D., Xu L. A High Linearity Detection Circuit with Constant Detection Voltage for Resistive Gas Sensor. Proceedings of the 2019 IEEE 4th Advanced Information Technology, Electronic and Automation Control Conference (IAEAC).

[28] Xie D., Liu R., Adedokun G., Xu L., Wu F. A Novel Low Power Hexagonal Gas Sensor Cell for Multi-Channel Gas Detection. Proceedings of the 2021 IEEE 34th International Conference on Micro Electro Mechanical Systems (MEMS).

[29] Xie D., Chen D., Peng S., Yang Y., Xu L., Wu F. (2019). A Low Power Cantilever-Based Metal Oxide Semiconductor Gas Sensor. IEEE Electron Device Lett..

[30] Sokolovskij R., Zhang J., Zheng H., Li W., Jiang Y., Yang G., Yu H., Sarro P.M., Zhang G. (2020). The Impact of Gate Recess on the H₂ Detection Properties of Pt-AlGaN/GaN HEMT Sensors. IEEE Sens. J..

[31] Fu H., Han Z., Li Y., Ma K. (2021). A Single-Pixel Sensor for Near-field Imaging Based on Startup Time of Oscillator. IEEE Microw. Wirel. Components Lett..

[32] Park K., Yeom S., Kim S.Y. (2020). Ultra-Low Power CMOS Image Sensor with Two-Step Logical Shift Algorithm-Based Correlated Double Sampling Scheme. IEEE Trans. Circuits Syst. I Regul. Pap..

[33] Liu Z., Liu Z., Ren E., Luo L., Wei Q., Wu X., Li X., Qiao F., Liu X.J. A 1.8mW Perception Chip with Near-Sensor Processing Scheme for Low-Power AIoT Applications. Proceedings of the 2019 IEEE Computer Society Annual Symposium on VLSI (ISVLSI).

[34] Park C., Zhao W., Park I., Sun N., Chae Y. (2021). A 51-pJ/Pixel 33.7-dB PSNR 4× Compressive CMOS Image Sensor with Column-Parallel Single-Shot Compressive Sensing. IEEE J. Solid-State Circuits.

[35] Hsu T.-H., Chen Y.-R., Liu R.-S., Lo C.-C., Tang K.-T., Chang M.-F., Hsieh C.-C. (2020). A 0.5-V Real-Time Computational CMOS Image Sensor with Programmable Kernel for Feature Extraction. IEEE J. Solid-State Circuits.

[36] Tian X., Liu Z., Guo C., Yang J., Chen J., Lyu S., Bi H., Qiao F., Wu X., Lu Y. (2021). Pressure Sensor Array with Low-Power Near-Sensor CMOS Chip for Human Gait Monitoring. IEEE Sens. Lett..

[37] Wang X., Meng X., Zhu Y., Ling H., Chen Y., Li Z., Hartel M.C., Dokmeci M.R., Zhang S., Khademhosseini A. (2021). A Sub-1-V, Microwatt Power-Consumption Iontronic Pressure Sensor Based on Organic Electrochemical Transistors. IEEE Electron Device Lett..

[38] Gardner E.L.W., de Luca A., Philippe J., Dragomirescu D., Udrea F. (2019). Thin-Film MOSFET-Based Pressure Sensor. IEEE Sens. Lett..

[39] Zhang C., Gallichan R., Lapshev S., Budgett D.M., McCormick D. A Low Power Capacitance to Frequency Converter in 180nm CMOS for an Implantable Capacitive Pressure Sensor. Proceedings of the 2019 IEEE Biomedical Circuits and Systems Conference (BioCAS).

[40] Lin Q., Xu J., Song S., Breeschoten A., Konijnenburg M., Van Hoof C., Tavernier F., Van Helleputte N. (2020). A 119dB Dynamic Range Charge Counting Light-to-Digital Converter for Wearable PPG/NIRS Monitoring Applications. IEEE Trans. Biomed. Circuits Syst..

[41] Song S., Konijnenburg M., Van Wegberg R., Xu J., Ha H., Sijbers W., Stanzione S., Biswas D., Breeschoten A., Vis P. (2019). A 769 μW Battery-Powered Single-Chip SoC with BLE for Multi-Modal Vital Sign Monitoring Health Patches. IEEE Trans. Biomed. Circuits Syst..

[42] Lin B., Ma Z., Atef M., Ying L., Wang G. (2021). Low-Power High-Sensitivity Photoplethysmography Sensor for Wearable Health Monitoring System. IEEE Sens. J..

[43] Huang Y.-J., Tzeng T.-H., Lin T.-W., Huang C.-W., Yen P.-W., Kuo P.-H., Lin C.-T., Lu S.-S. (2014). A Self-Powered CMOS Reconfigurable Multi-Sensor SoC for Biomedical Applications. IEEE J. Solid-State Circuits.

[44] Boukhayma A., Barison A., Haddad S., Caizzone A. (2021). Earbud-Embedded Micro-Power mm-Sized Optical Sensor for Accurate Heart Beat Monitoring. IEEE Sens. J..

[45] Qoutb A.G., Friedman E.G. Spintronic/CMOS-Based Thermal Sensors. Proceedings of the 2020 IEEE International Symposium on Circuits and Systems (ISCAS).

[46] Azam A., Bai Z., Walling J.S. (2021). An Ultra-Low Power CMOS Integrated Pulse-Width Modulated Temperature Sensor. IEEE Sens. J..

[47] Zemmour H., Baudoin G., Hamouda C., Diet A., Biancheri-Astier M. Impact of soil on UWB buried antenna and communication link in IR-UWB WUSN applications. Proceedings of the 2015 European Radar Conference (EuRAD).

[48] Zemmour H., Baudoin G., Diet A. (2017). Soil Effects on the Underground-to-Aboveground Communication Link in Ultrawideband Wireless Underground Sensor Networks. IEEE Antennas Wirel. Propag. Lett..

[49] Alkhalifeh K., Hislop G., Ozdemir N.A., Craeye C. (2016). Efficient MoM Simulation of 3-D Antennas in the Vicinity of the Ground. IEEE Trans. Antennas Propag..

[50] Han D., Polycarpou A.C., Balanis C.A. (2001). Ground effects for VHF/HF antennas on helicopter airframes. IEEE Trans. Antennas Propag..

[51] Varghese B., John N.E., Sreelal S., Gopal K. (2016). Design and Development of an RF Energy Harvesting Wireless Sensor Node (EH-WSN) for Aerospace Applications. Procedia Comput. Sci..

[52] Din M.S.U., Rehman M.A.U., Ullah R., Park C.-W., Kim B.S. (2020). Towards Network Lifetime Enhancement of Resource Constrained IoT Devices in Heterogeneous Wireless Sensor Networks. Sensors.

[53] Liu T., Qu X., Tan W., Cheng Y. (2019). An Energy Efficient Cooperative Communication Scheme in Ambient RF Powered Sensor Networks. IEEE Access.

[54] Caselli M., Ronchi M., Boni A. (2020). Power Management Circuits for Low-Power RF Energy Harvesters. J. Low Power Electron. Appl..

[55] Nguyen T.D., Khan J.Y., Ngo D.T. (2017). Energy harvested roadside IEEE 802.15.4 wireless sensor networks for IoT applications. Ad Hoc Netw..

[56] Nguyen T.D., Khan J.Y., Ngo D.T. An adaptive MAC protocol for RF energy harvesting wireless sensor networks. Proceedings of the 59th IEEE Global Communications Conference, GLOBECOM.

[57] Grossi M. (2021). Energy Harvesting Strategies for Wireless Sensor Networks and Mobile Devices: A Review. Electronics.

